# Maternal prenatal nut and seafood consumption and child neuropsychological function from 4 to 15 years of age: a population-based cohort study

**DOI:** 10.1016/j.ajcnut.2025.04.032

**Published:** 2025-05-05

**Authors:** Ariadna Pinar-Martí, Nicolas Ayala-Aldana, Marina Ruiz-Rivera, Nerea Lertxundi, Mikel Subiza-Pérez, Llúcia González-Safont, Jesús Vioque, Isolina Riaño-Galán, Cristina Rodríguez-Dehli, Lucía Iglesias-Vázquez, Victoria Arija, Silvia Fernández-Barrés, Dora Romaguera, Vicenç Pascual-Rubio, Albert Fabregat-Sanjuan, Darren Healy, Xavier Basagaña, Martine Vrijheid, Mònica Guxens, Maria Foraster, Jordi Julvez

**Affiliations:** 1 Clinical and Epidemiological Neuroscience (NeuroÈpia), Institut d’Investigació Sanitària Pere Virgili, Reus, Spain; 2 ISGlobal, Institut de Salut Global de Barcelona-Campus MAR, PRBB, Barcelona, Spain; 3 Departament de Medicina i Ciències de la Vida (MELIS), Universitat Pompeu Fabra, Barcelona, Spain; 4 Department of Clinical and Health Psychology and Research Methods, University of the Basque Country UPV/EHU, Leioa, Spain; 5 Group of Environmental Epidemiology and Child Development, Biogipuzkoa Health Research Institute, San Sebastian, Spain; 6 Centro de Investigación Biomédica en Red de Epidemiología y Salud Pública, Instituto de Salud Carlos III, Madrid, Spain; 7 Bradford Institute for Health Research, Bradford, United Kingdom; 8 Unidad Mixta de Investigación en Epidemiología, Ambiente y Salud. Fundación para el Fomento de la Investigación Sanitaria y Biomédica de la Comunidad Valenciana -Universitat Jaume I- Universitat de València, Valencia, Spain; 9 Fundación para el Fomento de la Investigación Sanitaria y Biomédica de la Comunidad Valenciana, Valencia, Spain; 10 Instituto de Investigación Sanitaria y Biomédica de Alicante, Universidad Miguel Hernández, Alicante, Spain; 11 Facultad de Medicina y Ciencias de la Salud, Universidad de Oviedo, Oviedo, Spain; 12 Instituto de Investigación Sanitaria del Principado de Asturias, Oviedo, Spain; 13 Endocrinología Pediátrica, Hospital Universitario Central de Asturias, Oviedo, Spain; 14 Servicio de Pediatría, Hospital San Agustín, Avilés, Asturias, Spain; 15 Nutrition and Mental Health (NUTRISAM) Research Group, Universitat Rovira i Virgili, Reus, Spain; 16 Institut d'Investigació Sanitària Pere Virgili, Tarragona, Spain; 17 Collaborative Group on Lifestyles, Nutrition, and Tobacco (CENIT), Institut d'Investigació en Atenció Primària IDIAP Jordi Gol. Institut Català de la Salut, Reus, Spain; 18 Institut d'Investigació en Atenció Primària IDIAP Jordi Gol. Institut Català de la Salut, Barcelona, Spain; 19 Agència de Salut Pública de Barcelona, Barcelona, Spain; 20 Hospital Sant Joan de Reus, Reus, Spain; 21 FUNCMAT, Mechanical Engineering Department, Universitat Rovira i Virgili, Tarragona, Spain; 22 Institute of Public Health and Clinical Nutrition, School of Medicine, Faculty of Health Sciences, University of Eastern Finland, Kuopio, Finland; 23 Department of Child and Adolescent Psychiatry/Psychology, Erasmus University Medical Center, Rotterdam, Netherlands; 24 PHAGEX Research Group, Blanquerna School of Health Science, Universitat Ramon Llull, Barcelona, Spain

**Keywords:** maternal diet, nuts and seafood, omega-3 fatty acids, pregnancy, neuropsychological development

## Abstract

**Background:**

Understanding the role of maternal diet in early brain development is critical, as pregnancy represents a period of significant vulnerability and growth for the developing brain.

**Objectives:**

This study aims to assess the association between maternal nuts, total seafood, and large fatty fish consumption during pregnancy and offspring neuropsychological function ≤15 y, considering the potential mediation of omega-3 fatty acids.

**Methods:**

This study was part of The Spanish Childhood and Environment birth cohort, following 1737 mother–child pairs from pregnancy to age 15. Maternal diet was evaluated using a semiquantitative food frequency questionnaire, whereas children’s neuropsychological function was measured through standardized computer-based tests. Attention (hit reaction time and its variability, HRT and HRT-SE) was measured with the Conners’ Kiddie Continuous Performance Test and the Attention Network Test. Working memory (detectability in 2-back, d2′, and 3-back tasks, d3′) was evaluated using the N-back task. Fluid intelligence was assessed with Raven’s Progressive Matrices and the Test of Primary Mental Abilities. Linear mixed-effects regression models assessed the association of nuts, seafood and large fatty fish with neuropsychological outcomes, whereas generalized structural equation modeling was used for mediation analyses.

**Results:**

Higher maternal nut consumption was significantly linked to improved attention [HRT-SE *β* = –0.05, 95% confidence interval (CI): –0.09, –0.00] and working memory (d2′ *β* = 0.05, 95% CI: 0.00, 0.09, and d3′ *β* = 0.06, 95% CI: 0.02, 0.11) in offspring. Greater consumption of large fatty fish was associated with better attention (HRT-SE *β* = –0.06, 95% CI: –0.10, –0.02; and HRT *β* = –0.04, 95% CI: –0.08, –0.00), and fluid intelligence (*β* = 0.08, 95% CI: 0.02, 0.13). Omega-3 fatty acids mediated 8%–14% of these effects on attention.

**Conclusions:**

Maternal diet at pregnancy and omega-3 intake may support long-term cognitive development in children and adolescents.

## Introduction

Nutrition is crucial for brain development, particularly during key periods of life when the brain experiences a peak of maturation [1]. Most brain development occurs during pregnancy, and much of its final structure is formed before the age of 3 [2]. However, several brain regions continue to develop during childhood and throughout adolescence [3]. The prefrontal cortex is essential for cognitive control and the modulation of higher-level abilities, making it crucial for cognitive functions like attention, working memory, and fluid intelligence [4,5]. Its development starts in the fetal stages, but it fully matures around the early twenties, undergoing substantial structural and functional changes during adolescence [3,6].

The brain is rich in long-chain omega-3 PUFAs, indicating their significant role in the development and function of brain structures and networks [7,8]. DHA (22:6n-3) is the primary omega-3 PUFA found in the brain, with a particularly high concentration in the prefrontal cortex [9]. The levels of DHA in bodily tissues are closely linked to dietary intake, primarily from fatty fish [10]. During pregnancy and lactation, developing infants rely on maternal dietary omega-3 supply [7,11]. Thus, nutritional deficiencies during this critical period can have long-term repercussions on the offspring’s developing brain [2,12].

Indeed, there is abundant evidence that seafood consumption during pregnancy and lactation is related to neuropsychological improvements in children [13,14]. However, the role of maternal dietary PUFAs on child neuropsychological development in later life has hardly been explored [15]. Similarly, research on nut consumption and brain neurodevelopment is scarce. Nuts are rich in plant-derived omega-3 fatty acid alpha-linolenic acid (ALA; 18:3n-3), the precursor for DHA [16]. Although it was previously believed that ALA influenced brain function and plasticity indirectly through its conversion to DHA, some clinical and mice studies have indicated that ALA alone can also have beneficial effects [[17], [18], [19]]. A previous study in our birth cohort found that nut intake during pregnancy was associated with long-term child neuropsychological development ≤8 y of age [20]. Thus, it would be interesting to elucidate whether these longitudinal associations persist in older ages. Moreover, most studies focus on the impact of nut or fish consumption on cognition at a single time point, rather than across multiple developmental stages.

Given all of the above, the aim of the present study is to determine whether a high prenatal intake of nuts and seafood is positively associated with improvements in offspring’s neuropsychological function over time (≤15 y), particularly in attention, working memory, and fluid intelligence. Furthermore, we will assess whether cord-blood omega-3 fatty acid levels, reflecting the maternal supply during pregnancy and linked to the consumption of these foods, have a mediating role in this association.

## Methods

### Study design and participants

**FIGURE 1:**
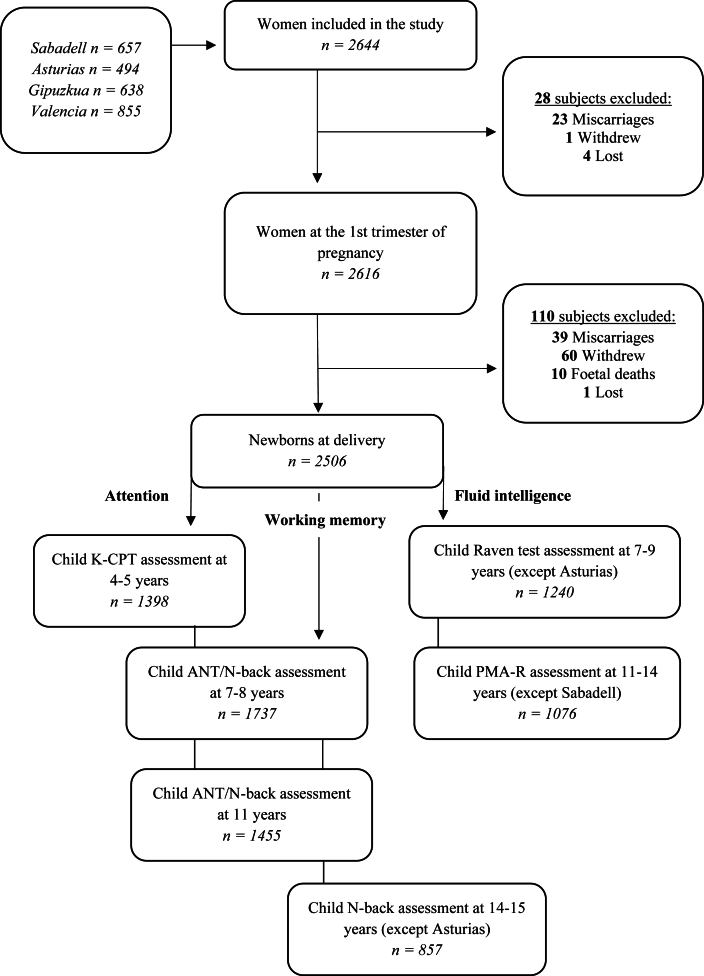
Flowchart of the population of the study: Infancia y Medio Ambiente (INMA) Project, 2004–2019. ANT, Attention Network Test; KCP-T, Conners' Kiddie Continuous Performance Test; PMA-R, Primary Mental Abilities—Reasoning.

This study is based on The Spanish Childhood and Environment [Infancia y Medio Ambiente (INMA)] project, a population-based birth cohort with the aim of studying the developmental effects of major environmental pollutants in air, water, and diet during pregnancy and early life. The project was established between 2003 and 2008 in various regions of Spain. The present study is focused on 4 regions: Asturias, Gipuzkoa (Basque Country), Sabadell (Catalonia), and Valencia. A total of 2644 eligible women were recruited during first-trimester prenatal visits if they met the inclusion criteria (≥16 y old, singleton pregnancy, and planned delivery at the reference hospital). Exclusion criteria included communication handicaps, fetal malformations, and assisted conception. After exclusion, a total of 2506 pregnant women were followed up through delivery, and their children were enrolled at birth and followed up until the age of 14–16 y, completing different neuropsychological tests. Main analyses included between 857 and 1737 children, depending on the neuropsychological test, cohorts, and visits included in the analyses (Figure 1). Losses are attributable to follow-up, missing data on some variables, and the availability of neuropsychological tests by cohort. Each cohort varies slightly in follow-up time points, but all included assessments at key stages: first and third trimesters of pregnancy, birth, and ages 4, 7, 11, and 14–16 y. The project is ongoing, with the 18-y follow-up currently underway. All participants provided written informed consent at recruitment and follow-ups. Further information has been published elsewhere [21].

### Ethics statement

Ethical approval for data collection and analysis for the INMA project was given by hospital and institutional ethics committees in each region. The reference hospitals were: Sabadell (Hospital de Sabadell—Corporació Parc Taulí or Hospital de Terrassa), Valencia (Hospital La Fe), Asturias (Hospital San Agustín in Avilés), and Gipuzkoa (Hospital de Zumarraga in Gipuzkoa).

### Dietary data: nuts and seafood consumption

A semiquantitative food frequency questionnaire (FFQ) of 101 food items was used to assess the usual daily intake of foods and nutrients during the first trimester of pregnancy (10–13 wk), covering usual dietary intake from preconception through the third month of pregnancy, and was adapted and validated for Spanish pregnant women [22]. Women reported their usual dietary intake from the last menstruation to the first prenatal visit, using reference portions and 9 frequency categories ranging from never/less than once a month to >6 times per day. The same FFQ was administered in the third trimester of pregnancy (28–32 wk) to assess typical dietary intake from the fourth to the seventh months of pregnancy. However, for this study, we only use dietary data from the first trimester, as previous research in the same cohort has found stronger associations with first-trimester dietary data [20,23]. Moreover, the correlation between first- and third-trimester dietary data was relatively high for self-reported information (Spearman’s rho = 0.49 for fish and 0.39 for nuts). The questionnaire included 1 item related to nut consumption, covering walnuts, almonds, peanuts, pine seeds and hazelnuts (1 portion of 30 g), and 8 seafood items: large fatty fish (tuna, swordfish, albacore), small fatty fish (mackerel, sardines, anchovies, salmon), canned tuna, lean fish (hake, sole, bream), fried fish, shellfish (shrimp, prawns, lobster, crab, clams, mussels, oysters, squid, octopus, cuttlefish), processed fish (for example, surimi), and smoked or salted fish. The responses of each item were converted to mean weekly intakes in grams (g) and, in the case of seafood, were then summed to compute the total and seafood subtypes (in g/wk). For seafood consumption, we considered 2 main exposures to include in the models: total seafood intake and intake of large fatty fish (because it is richer in omega-3s). Intakes were adjusted for energy intake using the residual method [24] and analyzed in tertile categories of weekly grams for total nut, seafood, and large fatty fish. Further information on covariable information can be found in Supplemental Methods (covariable information).

### Omega-3 determinations

Whole blood samples were collected via venipuncture of cord vessels before placental delivery. Samples were processed, aliquoted into 1 mL portions, and stored at −80°C until analysis. In a subsample of cord plasma (*n* = 948), PUFA methyl esters were prepared and extracted using a validated method [25]. PUFAs were then separated and quantified by fast-gas chromatography, using a Shimadzu GC-2010 gas chromatograph with a flame ionization detector (Shimadzu). Fatty acid methyl esters were separated on a Varian VF-23 ms capillary column (10 m × 0.10 mm i.d. × 0.10 mm film thickness) with a high cyanopropyl stationary phase (Varian). Helium served as the carrier gas, and the injector operated in split mode (1:30). The temperature program included an initial temperature of 120°C, a first ramp of 35°C/min to 175°C (held for 1.5 min), and a second ramp of 20°C/min to 250°C. Injector and detector temperatures were set at 250°C and 300°C, respectively. Data were acquired and processed using Shimadzu-ChemStation software [26]. Individual ALA, EPA and DHA were measured and expressed in percentage of the total fatty acids.

### Neuropsychological assessments

Several internationally validated and standardized computer-based neuropsychological tests were used to assess neuropsychological outcomes in children. Attention function was assessed at a mean age of 4.9 y (SD = 0.6) using the Conners’ Kiddie Continuous Performance Test (K-CPT) and at ages 7.5 (SD = 0.7) and 11.1 (SD = 0.7) with the Attention Network Test (ANT). Both tests examine sustained attention based on reaction times (RT) to target stimuli, but K-CPT takes only half the time (7.5 min) to complete, making it more appropriate for younger children. The outcomes selected were hit reaction time (HRT) and hit reaction time SE (HRT-SE), which measure mean RT and its variability for correct responses, respectively. Outcomes are measured in milliseconds (ms) and lower scores indicate better attention performance. An extensive description is published elsewhere [27]. The second primary outcome, working memory, was assessed using the N-back task at ages 7.5 (0.7), 11.1 (0.7), and 15 (0.8) y [28]. Participants identified whether the current stimulus matched 1 presented *n* trials earlier (1-, 2-, 3-, or 4-back). The selected outcomes were detectability (*d*’) for the 2-back and 3-back conditions, with higher scores indicating better performance. Finally, fluid intelligence was assessed using the Raven test (Raven’s Progressive Matrices) [29] at age 9.2 (SD = 1.3) and the Test of Primary Mental Abilities (the Spanish adaptation) [30] at age 14.5 (SD = 1.2). The total score reflects the number of correct responses, with higher scores indicating better performance. All outcomes were standardized using z-scores to ensure consistency across tests.

### Statistical analysis

Descriptive statistics were used to display the baseline characteristics of the study population according to nuts and fish consumption at the first trimester of pregnancy. Categorical variables are expressed as percentages, and continuous variables are expressed as means (SD).

The primary analysis was performed using linear mixed-effect models to assess the association between maternal consumption of nuts, total seafood, and large fatty fish with offspring attention, working memory, and fluid intelligence up to age 15. Follow-up scores from neuropsychological tests were used as the outcome variable (continuous, for each outcome separately). Maternal intake of nuts, total seafood, and large fatty fish were treated as the main explanatory variables, categorized into tertiles of low, medium, and high intake. Cohort was used as a random effect, with individuals as random intercepts and treating visit number as nested within individuals, ensuring that each participant's multiple observations over time were correctly accounted for. The main confounders were selected according to previous scientific knowledge and then built a Directed Acyclic Graph (DAG) model (Supplemental Figure 1). Models were adjusted for maternal energy intake (calories, kcal/d), child’s sex, and children’s age at each visit (years) as default adjustment variables, and were further adjusted by mother’s age at delivery (years), omega-3 supplementation (yes/no), modified relative Mediterranean diet score (rMed, in tertiles), maternal verbal intelligence quotient (IQ, continuous, standardized as mean=10, SD = 3), maternal education (primary school or less, secondary, university), and occupational social class (I/II: managers/technicians, III: nonmanual, and IV/V: manual) according to DAG’s minimal sufficient adjustment set. Linear mixed models handle missing data by assuming that repeated measurements from the same participant are correlated [31]. This approach allows the models to maximize the use of all available data, even when some measures are missing at certain time points.

The secondary analysis involved mediation analyses, performed using generalized structural equation modeling (gsem), to estimate the proportion of the effect of maternal nut, seafood and large fatty fish consumption during pregnancy on child neuropsychological function mediated by omega-3 fatty acids related to the consumption of these foods (sum of ALA, EPA and DHA) measured in cord-blood plasma at the time of delivery. As gsem does not allow random-effects, cohort and visit were used as additional adjustment variables. Total seafood consumption was not tested for mediation because no statistical significance was found in any main analysis. Associations between the outcomes and the mediator were tested to ensure the criteria for performing a mediation analysis were met. HRT-SE and N-back d3′ were the only outcomes that showed a significant association with omega-3 (*P* = 0.020 and *P* = 0.025, respectively). Additionally, correlation analyses revealed a significant association between nut and large fatty fish intake with the mediator (Supplemental Table 1). Mediation was deemed significant only if the indirect effect (NIE) reached statistical significance [32].

Finally, several sensitivity analyses on the association of nuts and large fatty fish consumption at the first trimester of pregnancy and child neurocognitive outcomes were carried out with additional adjustments to ensure that the observed associations were not confounded by external factors that could influence neurodevelopment. These factors included: *1*) child’s nuts intake at ages 4–5 y and 7–8 y; *2*) child’s fish intake at ages 4–5 y and 7–8 y; *3*) breastfeeding duration in weeks; *4*) cord-blood mercury (only for large fatty fish); *5*) maternal serum hexachlorobenzene levels (only for large fatty fish); *6*) maternal serum dichlorodiphenyl dichloroethylene levels (only for large fatty fish); *7*) maternal serum dichlorodiphenyltrichloroethane levels (only for large fatty fish); *8*) maternal serum polychlorinated biphenyls (PCBs, sum of 118, 153, 138, 180) levels (only for large fatty fish).

Calculations were corrected for multiplicity using the Benjamini–Hochberg method. Because no universal false discovery rate (FDR) significance threshold has been established, a cut-off (*α*) of 0.05 was applied. FDR correction was only applied to *P*-for-trends. The nominal statistical significance was set at *P* < 0.05 level (2-sided) in all models. Statistical analyses were conducted using STATA 16 statistical software package (StataCorp LLC).

## Results

### Descriptive analysis

The median maternal nut consumption during the first trimester of pregnancy was 17.50 g/wk (IQR = 0–46.24) and the median of the highest tertile of nut consumption was 73.41 g/wk (IQR = 46.24–124.21) (Table 1). Women who reported a higher intake of nuts showed a lower prepregnancy BMI, higher verbal IQ, and higher educational level. Likewise, they reported a lower energy intake (kcal/d) and scored higher in the rMed. Children whose mothers had a higher intake of nuts had a longer breastfeeding duration and higher cord-blood levels of mercury, EPA, and DHA. In contrast, ALA cord-blood levels were lower in the highest tertile of nut consumption (Table 1). Participants showed similar characteristics across tertiles of seafood consumption (Supplemental Table 2). The median maternal seafood consumption during the first trimester of pregnancy was 445.70 g/wk (IQR = 299.72–630.81) and the median of the highest tertile was 727.12 g/wk (IQR = 630.81; 884.78). The exposure variables were positively correlated with cord-blood concentrations of omega-3 fatty acids and Hg (Supplemental Table 1). Child neuropsychological scores at each visit according to maternal nut and large fatty fish consumption are shown in Supplemental Tables 3 and 4.

**TABLE 1:** Baseline characteristics of the study participants, shown by tertiles of maternal nut consumption in the first trimester of pregnancy.

	Total (*N* = 2585)	Tertiles of maternal nut intake		
		Low (*n* = 861)^1^	Medium (*n* = 862)^1^	High (*n* = 862)^1^
Maternal characteristics^2^				
Nut intake in g/wk, median (IQR)	17.50 (0.00–46.24)	0.00 (0.00–0.00)	17.44 (11.68–24.27)	73.41 (46.24–124.21)
Age in years, mean (SD)	30.59 (4.37)	30.35 (4.55)	30.59 (4.33)	30.85 (4.23)
Cohort location				
Sabadell	654 (25.30)	238 (27.64)	222 (25.75)	194 (22.51)
Asturias	482 (18.65)	188 (21.84)	128 (14.85)	166 (19.26)
Valencia	822 (31.80)	270 (31.36)	341 (39.56)	211 (24.48)
Gipuzkoa	627 (24.26)	165 (19.16)	171 (19.84)	291 (33.76)
Prepregnancy BMI (in kg/m^2^)	23.58 (4.32)	24.45 (4.83)	23.42 (4.40)	22.86 (3.49)
IQ, mean (SD)	9.94 (2.97)	9.65 (2.98)	10.04 (2.97)	10.12 (2.93)
Education				
Primary school or less	648 (25.11)	245 (28.52)	234 (27.18)	169 (19.63)
Secondary school	1066 (41.30)	358 (41.68)	372 (43.21)	336 (39.02)
University or more	867 (33.59)	256 (29.80)	255 (29.62)	356 (41.35)
Country of birth				
Spain	2364 (91.56)	767 (89.19)	787 (91.51)	810 (93.97)
Other	218 (8.44)	93 (10.81)	73 (8.49)	52 (6.03)
Smoking during pregnancy	1338 (54.97)	455 (56.24)	477 (58.38)	406 (50.25)
Alcohol consumption during pregnancy	789 (30.52)	230 (26.71)	286 (33.18)	273 (31.67)
rMED				
Low	1085 (41.97)	371 (43.09)	401 (46.52)	313 (36.31)
Medium	748 (28.94)	244 (28.34)	245 (28.42)	259 (30.05)
High	752 (29.09)	246 (28.47)	216 (25.06)	290 (33.64)
Energy intake in kcals/d, mean (SD)	2120.01 (556.64)	2026.08 (519.08)	2346.10 (584.51)	1987.73 (491.54)
Omega-3 supplementation	131 (5.07)	37 (4.30)	46 (5.34)	48 (5.57)
Child characteristics^2^				
Sex, female	1202 (48.43)	388 (46.86)	407 (49.15)	407 (49.27)
Birthweight in g				
<3000	642 (24.84)	217 (25.20)	214 (24.83)	211 (24.48)
3000–3500	1133 (43.83)	373 (43.32)	396 (45.94)	364 (42.23
>3500	810 (21.33)	217 (31.48)	252 (29.23)	287 (33.29)
Breastfeeding time				
None	340 (13.15)	140 (16.26)	106 (12.30)	94 (10.90)
0–16 wk	582 (22.51)	218 (25.32)	191 (22.16)	173 (20.07)
16–24 wk	363 (14.04)	121 (14.05)	112 (12.99)	130 (15.08)
>24 wk	1300 (50.29)	382 (44.37)	453 (52.55)	465 (53.94)
Mercury cord-blood levels, mean (SD)^3^	0.91 (0.34)	0.90 (0.35)	0.90 (0.35)	0.94 (0.32)
Omega-3 fatty acids umbilical cord-blood levels^4^				
ALA in %, mean (SD)	0.10 (0.10)	0.11 (0.12)	0.10 (0.09)	0.09 (0.09)
DHA in %, mean (SD)	5.11 (1.66)	4.86 (1.52)	5.01 (1.62)	5.46 (1.78)
EPA in %, mean (SD)	0.23 (0.18)	0.22 (0.19)	0.22 (0.14)	0.27 (0.21)

Abbreviations: ALA, α-linolenic acid; IQ, intelligence quotient; rMed, relative Mediterranean diet score.

1 Some of totals may not match the total number of subjects due to missings.

2 Unless otherwise indicated, data are expressed as number (percentage) of participants. Percentages have been rounded and may not total 100.

3 Cord-blood mercury (log-transformed) was only measured in a subset of participants (*N* = 1872), *n* = 603 for the first tertile, *n* = 628 for the second tertile, and *n* = 641 for the third tertile.

4 Omega-3 fatty acids were only measured in a subset of participants (*N* = 948), *n* = 310 for the first tertile, *n* = 326 for the second tertile, and *n* = 312 for the third tertile of maternal nut intake.

### Primary analysis

The models for the association of nut consumption during pregnancy with offspring neuropsychological function ≤15 y of age are shown in Table 2. The same models with large fatty fish and total seafood intake as the exposure are shown in TABLE 3, TABLE 4. Children whose mothers had a higher nut intake during pregnancy showed greater development of attention and working memory than the children of mothers who had a lower nut intake (Table 2). HRT-SE was lower across tertiles of nut consumption (*β* = –0.05, 95% CI: –0.09, –0.00, *P* for trend = 0.041). For working memory, both N-back outcomes (d2′ and d3′) had a higher score across tertiles of nut consumption (d2′ *β* = 0.05, 95% CI: 0.00, 0.09, *P* for trend = 0.043; and d3′ *β* = 0.06, 95% CI: 0.02, 0.11, *P* for trend = 0.007). No association with fluid intelligence was observed. In terms of total seafood consumption, no significant differences were found for any neuropsychological outcome (Table 4). However, higher maternal large fatty fish consumption showed greater cognitive function in the offspring for attention and fluid intelligence (Table 3). HRT-SE and mean HRT were lower across tertiles (*β* = –0.06, 95% CI: –0.10, –0.02, *P* for trend = 0.004; and *β* = –0.04, 95% CI –0.08, –0.00, *P* for trend = 0.032, respectively). For fluid intelligence, the total score was higher across tertiles of large fatty fish consumption (*β* = 0.08, 95% CI: 0.02, 0.13, *P* for trend = 0.006). Notably, in all models for large fatty fish consumption, the association was greater in the second tertile than in the highest tertile. Such is the case that, although we did not find significant differences in general for working memory, we did find a slight improvement for the d3’-back model for the second tertile of large fatty fish consumption compared with the lowest (*β* = 0.10, 95% CI: 0.01, 0.20, *P* = 0.034).

**TABLE 2:** Association between nut consumption during pregnancy and offspring neuropsychological function ≤15 y of age.

Neuropsychological outcome^1^	*N*	Maternal nut intake in the first trimester	*β* coeff.^2^	95% CI	*P* value
Attention^3^ ANT (HRT-SE)	1688	Lowest tertile	Ref.		
		Middle tertile	0.05	–0.05, 0.14	0.329
		Higher tertile	–0.09	–0.18, –0.01	0.041
		Tertiles in continuous	–0.05	–0.09, –0.00	0.041
(HRT mean)	1688	Lowest tertile			
		Middle tertile	0.06	–0.03, 0.15	0.163
		Higher tertile	–0.05	–0.14, 0.03	0.209
		Tertiles in continuous	–0.03	–0.07, 0.02	0.226
Working memory^4^ N-back (d2′)	1565	Lowest tertile	Ref.		
		Middle tertile	–0.00	–0.09, 0.09	0.962
		Higher tertile	0.09	0.00, 0.18	0.045
		Tertiles in continuous	0.05	0.00, 0.09	0.043
(d3′)	1557	Lowest tertile	Ref.		
		Middle tertile	0.06	–0.04: 0.15	0.250
		Higher tertile	0.12	0.03, 0.21	0.007
		Tertiles in continuous	0.06	0.02, 0.11	0.007
Fluid intelligence^4^ (PMA-R and Raven test)	1297	Lowest tertile	Ref.		
		Middle tertile	–0.04	–0.16, 0.08	0.472
		Higher tertile	0.10	–0.02, 0.22	0.090
		Tertiles in continuous	0.05	–0.01, 0.11	0.085

Abbreviations: ANT, Attention Network Test; CI, confidence interval; HRT-SE, hit reaction time—SE; PMA-R, Primary Mental Abilities—Reasoning.

Median (IQR) of nut intake in each tertile in g/wk: lowest = 0.00 (0.00–0.00); middle = 17.44 (11.68–24.27); highest = 73.41 (46.24–124.21).

1 Neuropsychological outcome scores were standardized as *z*-scores.

2 Data were calculated using a linear mixed model with cohort and individuals as random-effects, visit as random slope, and adjusted for mother’s age at delivery, children’s sex and age on each visit, mother’s energy intake, maternal omega-3 supplementation during pregnancy, maternal IQ, rMed score (without nuts), maternal education and maternal social class.

3 Lower scores indicate better performance.

4 Higher scores indicate better performance.

**TABLE 3:** Association between large fatty fish consumption during pregnancy and offspring neuropsychological function ≤15 y of age.

Neuropsychological outcome^1^	*N*	Maternal fish intake in the first trimester	*β* Coeff.^2^	95% CI	*P* value
Attention^3^ ANT (HRT-SE)	1688	Lowest tertile	Ref.		
		Middle tertile	–0.14	–0.24, –0.04	0.004
		Higher tertile	–0.12	–0.20, –0.03	0.006
		Tertiles in continuous	–0.06	–0.10, –0.02	0.004
(HRT mean)	1688	Lowest tertile			
		Middle tertile	–0.10	–0.19, –0.07	0.035
		Higher tertile	–0.08	–0.16, –0.00	0.045
		Tertiles in continuous	–0.04	–0.08, –0.00	0.032
Working memory^4^ N-back (d2′)	1565	Lowest tertile	Ref.		
		Middle tertile	0.02	–0.07, 0.12	0.639
		Higher tertile	0.05	–0.03, 0.14	0.221
		Tertiles in continuous	0.03	–0.02, 0.07	0.222
(d3′)	1557	Lowest tertile	Ref.		
		Middle tertile	0.10	0.01, 0.20	0.034
		Higher tertile	0.02	–0.06, 0.11	0.595
		Tertiles in continuous	0.01	–0.03, 0.06	0.501
Fluid intelligence^4^ (PMA-R and Raven test)	1297	Lowest tertile	Ref.		
		Middle tertile	0.18	0.06, 0.31	0.003
		Higher tertile	0.15	0.04, 0.26	0.009
		Tertiles in continuous	0.08	0.02, 0.13	0.006

Abbreviations: ANT, Attention Network Test; CI, confidence interval; HRT-SE, hit reaction time—SE; PMA-R, Primary Mental Abilities—Reasoning.

Median (IQR) of large fatty fish intake in each tertile in g/wk: lowest = 0.00 (0.00–0.00); middle = 24.36 (20.99–29.20); highest = 56.98 (46.94–124.28).

1 Neuropsychological outcome scores were standardized as *z*-scores.

2 Data were calculated using a linear mixed model with cohort and individuals as random-effects, visit as random slope, and adjusted for mother’s age at delivery, children’s sex and age on each visit, mother’s energy intake, maternal omega-3 supplementation during pregnancy, maternal IQ, rMed score (without fish), maternal education and maternal social class.

3 Lower scores indicate better performance.

4 Higher scores indicate better performance.

**TABLE 4:** Association between total seafood consumption during pregnancy and offspring neuropsychological function ≤15 y of age.

Neuropsychological outcome^1^	*N*	Maternal fish intake in the first trimester	*β* Coeff.^2^	95% CI	*P* value
Attention^3^ ANT (HRT-SE)	1688	Lowest tertile	Ref.		
		Middle tertile	–0.07	–0.16, 0.02	0.146
		Higher tertile	–0.05	–0.14, 0.04	0.325
		Tertiles in continuous	–0.02	–0.07, 0.02	0.338
(HRT mean)	1688	Lowest tertile	Ref.		
		Middle tertile	–0.06	–0.14, 0.03	0.191
		Higher tertile	–0.03	–0.11, 0.06	0.528
		Tertiles in continuous	–0.01	–0.06, 0.03	0.548
Working Memory^4^ N-back (d2′)	1565	Lowest tertile	Ref.		
		Middle tertile	0.01	–0.08, 0.10	0.819
		Higher tertile	0.00	–0.09, 0.09	0.934
		Tertiles in continuous	0.00	–0.04, 0.05	0.940
(d3′)	1557	Lowest tertile	Ref.		
		Middle tertile	0.03	–0.06, 0.13	0.468
		Higher tertile	0.03	–0.06, 0.13	0.482
		Tertiles in continuous	0.02	–0.03, 0.06	0.492
Fluid intelligence^4^ (PMA-R and Raven)	1297	Lowest tertile	Ref.		
		Middle tertile	0.04	–0.08, 0.16	0.532
		Higher tertile	–0.03	–0.14, 0.09	0.661
		Tertiles in continuous	–0.01	–0.07, 0.04	0.632

Abbreviations: ANT, Attention Network Test; CI, confidence interval; HRT-SE, hit reaction time—SE; PMA-R, Primary Mental Abilities—Reasoning.

Median (IQR) of fish intake in each tertile in g/wk: lowest = 249.48 (168.77–299.50); middle = 445.66 (400.45–499.64); highest = 727.12 (630.81–884.78).

1 Neuropsychological outcome scores were standardized as *z*-scores.

2 Data were calculated using a linear mixed model with cohort and individuals as random-effects, visit as random slope, and adjusted for mother’s age at delivery, children’s sex and age on each visit, mother’s energy intake, maternal omega-3 supplementation during pregnancy, maternal IQ, rMed score (without fish), maternal education and maternal social class.

3 Lower scores indicate better performance.

4 Higher scores indicate better performance.

### Secondary analysis

Table 5 shows the direct and indirect effects of nut and large fatty fish intake during pregnancy (as tertiles in continuous) on children’s cognitive development mediated by omega-3 fatty acids (sum of DHA, EPA, and ALA). Linear mixed-effect models that were statistically significant (*P* for trend) were tested for mediation if the association between the outcome and omega-3 was statistically significant as well. Significant associations with omega-3 were found only for HRT-SE and d3′. For nuts, the NIE appears to be significant for attention, but not for working memory. Thus, the mediation of omega-3 for attention explains 14.41% of the total effect of nuts on ANT–HRT-SE. For large fatty fish, the mediator accounted for 8.51% of the total explained variance for ANT–HRT-SE.

**TABLE 5:** Direct and indirect effects of nut and large fatty fish intake during pregnancy on child’s cognitive development mediated by omega-3 fatty acids (EPA+DHA+ALA) cord-blood levels at delivery.

Neuropsychological outcomes^1^^,^^2^	*N*	NDE^3^ (95% CI)	NIE^4^ (95% CI)	TE^5^ (95% CI)	% of total effect mediated by omega-3^6^
Nuts maternal intake					
Attention (ANT–HRT-SE)	1577	–0.074 (–0.134, –0.014)	–0.013 (–0.014, –0.011)	–0.087 (–0.148, –0.026)	14.41
Working memory N-back (d3′)	1377	0.106 (0.041, 0.172)	0.009 (–0.009, 0.027)	0.115 (0.032, 0.198)	7.90
Large fatty fish maternal intake					
Attention (ANT–HRT-SE)	1577	–0.121 (–0.178, –0.064)	–0.011 (–0.013, –0.010)	–0.132 (–0.191, –0.074)	8.51

Abbreviations: ANT, Attention Network Test; CI, confidence interval; HRT-SE, hit reaction time—SE; NDE, natural direct effect; NIE, natural indirect effect; PMA-R, Primary Mental Abilities—Reasoning; TE, total effect.

1 Neuropsychological outcome scores were standardized as *z*-scores.

2 Data were calculated using generalized structural equation modeling, adjusting for cohort, mother’s age at delivery, children’s sex and age on each visit, mother’s energy intake, number of visit, maternal omega-3 supplementation during pregnancy, maternal IQ, rMed score (without nuts or fish), maternal education and maternal social class.

3 Maternal nut/large fatty fish intake → neuropsychological outcome.

4 Maternal l nut/large fatty fish intake → omega-3 cord-blood levels at delivery → neuropsychological outcome.

5 Combined natural direct effect and natural indirect effect.

6 Ratio of natural indirect effect to total effect.

### Sensitivity analysis and multiple testing correction

The sensitivity analyses, which adjusted for child nut and seafood intake between 4 and 8 y of age, breastfeeding duration, Hg cord-blood levels, and the concentrations of several OCs in maternal serum, did not show differences in the magnitude of the associations for any of the outcomes compared with the main analysis (Supplemental Tables 5–13).

After correcting *P* values for multiple testing, fatty fish consumption lost statistical significance with HRT, as did nut consumption with HRT-SE and d2′ (Supplemental Table 14). These results have a slight chance to be considered potential false-positive discoveries. However, nuts maintained a statistically significant association with d3′ (working memory), as did large fatty fish with HRT-SE (attention). Therefore, the only association to be considered with caution is nuts with attention, as no attention outcomes remain significant for nut consumption.

## Discussion

In this birth cohort study, children of mothers with higher nut or large fatty fish consumption during pregnancy presented better neuropsychological development up to age 15 compared with those with lower intake. Higher nut consumption was associated with improved attention and working memory in the offspring, though the attention findings should be interpreted with caution due to multiple testing corrections. Although total seafood intake showed no associations, higher large fatty fish consumption was associated with improved attention and fluid intelligence. Moreover, omega-3 fatty acid levels in cord-blood appeared to mediate the relationship between maternal nut and large fatty fish intake and children's attention development.

Previous literature on the relationship between nut consumption during pregnancy and neuropsychological function in the offspring is scarce, limited to 1 longitudinal study, conducted in the same population [20]. This previous study found a positive association of nut consumption during pregnancy with child attention at 8 y of age. Our findings indicate a potential association up to age 11, suggesting these associations may persist into later years. However, this result should be interpreted with caution due to the potential risk of being a false discovery. Notably, our study found a positive association with working memory in the offspring ≤15 y of age. Although studies on maternal nut consumption during pregnancy and offspring neuropsychological function are limited, most research has focused on its effects on cognitive decline in the elderly. For instance, 2 randomized interventional studies on a Mediterranean diet supplemented with nuts reported improvements in general cognitive function, including attention and working memory [33,34]. Although this suggests a link between nut consumption and working memory, further studies with repeated outcome measurements during neurodevelopment are needed.

Our results for fish consumption are in line with those from prior studies. Previous INMA studies showed a positive association of maternal fish consumption with child neuropsychological development at 5 and 8 y of age [23,35], the latter showing a strong association for attention. Similarly, in the present study, we observed a positive association with attention ≤11 y and fluid intelligence ≤15 y, showing a continuity of the association with fish consumption during pregnancy throughout childhood and adolescence. However, we only found associations for large fatty fish, whereas the previous study reported associations for total seafood intake, lean fish, and small fatty fish. This may be due to large fatty fish containing greater amounts of omega-3 DHA and EPA, which could result in a longer-lasting effect [36]. Contrary to the case of nuts, the relationship between fish consumption during pregnancy and neurodevelopment has been extensively studied, but outcome trajectory analyses are still lacking [14]. Most studies report positive associations with various cognitive outcomes, including intelligence (IQ) and attention [37,38]. Some studies, however, suggest an attenuation of the effect in the highest category of seafood intake, which aligns with our findings. One feasible hypothesis could be the neurotoxic effect of mercury, which accumulates more in large fatty fish compared with other fish types [39]. However, large fatty fish also have the highest content of omega-3 PUFAs [36]. The balance between mercury's harmful effects and the benefits of fish nutrients remains controversial and inconclusive [23,[40], [41], [42]]. Our sensitivity analysis indicated that adjusting for mercury cord-blood levels did not alter the association between large fatty fish and offspring neuropsychological function. Likewise, adjusting for other fish contaminants (that is, organochlorines and PCBs) did not affect the primary results. Overall, it appears that the deleterious effects of mercury and other pollutants may be counteracted by the “beneficial” nutrients in fish, such as PUFAs and selenium [[41], [42], [43]].

An important finding of our study is the mediation effect of omega-3 on the association of nuts and large fatty fish intake during pregnancy with attention in the offspring. Nuts, especially walnuts, are an important source of omega-3 ALA, whereas large fatty fish are an important source of DHA and EPA. During pregnancy, the placenta regulates fatty acid supply to the fetus, with a selective preference for DHA, followed by ALA [44]. EPA facilitates DHA transport by increasing the production of fatty acid transporter and binding proteins [45]. DHA accrues rapidly in neural tissues, aiding synapse and dendritic spine formation, especially in the forebrain [46], which is particularly important for executive functions like attentional control and working memory [4,47,48]. Moreover, omega-3 PUFAs are key for proper neurotransmission, particularly in the monoaminergic system, which plays a significant role in attention modulation [49,50]. Additionally, ALA helps reduce neuroinflammation and boosts brain-derived neurotrophic factor, essential for neural growth and plasticity, and thus memory [51,52].

Although we found no relationship of omega-3s with fluid intelligence or working memory, we did find a positive relationship with the parent foods of these fatty acids. Both nuts and fish have other beneficial nutrients for proper brain function and development. Nuts, particularly walnuts, contain antioxidants and anti-inflammatory compounds like polyphenols, selenium, and vitamin E, which help combat neuroinflammation and oxidative damage by enhancing antioxidant defense [53,54]. Fish, in turn, also contains high levels of selenium, iodine, and vitamin D. In addition to its significant antioxidant effect, selenium aids neurotransmission and modulates monoaminergic systems, whereas vitamin D influences neurotransmitters and neurotrophic factors [55,56]. Iodine is essential for the production of thyroid hormones, which play a vital role in the central nervous system development and neurotransmitters regulation [57]. In fact, iodine deficiency during pregnancy has been associated with substantial impairments in learning and intelligence in the offspring [[57], [58], [59], [60]].

This study has several strengths. First, its longitudinal design allows for drawing a certain degree of causal inferences on how perinatal nutrition influences long-term cognitive outcomes. The duration of the study, spanning from pregnancy to 15 y of age, provides a comprehensive evaluation of cognitive development over time and enables to assessment of associations with perinatal and early-life exposures. Further, our results remain robust across several sensitivity analyses, including adjustments for child’s postnatal diet, breastfeeding duration, mercury cord-blood levels, and maternal serum OC concentrations. The use of omega-3 biomarkers also provides a more objective understanding of the relationship between perinatal nutrition and cognitive development, potentially identifying specific nutrients that are most beneficial. Nevertheless, this study has some limitations. The use of an FFQ relies on self-reported data and can be subject to recall bias and inaccuracies, potentially overestimating associations. Despite efforts to control for confounding factors, residual confounding may persist due to unmeasured variables that could influence both perinatal nutrition and cognitive outcomes. Finally, although the findings suggest a positive association between maternal diet and neuropsychological development in Spain, caution is needed when generalizing these results to other populations or regions with different dietary patterns. Further research is needed to validate the robustness of these findings and confirm them across different populations and settings, especially regarding the role of omega-3 fatty acids. Future studies should also explore whether these results persist into later adolescence and examine the impact of age on these associations to determine when they are most prominent.

Overall, our findings suggest that higher maternal consumption of nuts and large fatty fish during pregnancy is associated with some improved aspects of neuropsychological function in children ≤15 y of age. Specifically, higher nut consumption was associated with better working memory development and possibly attention, whereas greater intake of large fatty fish was associated with enhanced attention and fluid intelligence. Notably, omega-3 levels seem to partially mediate the positive associations with attention, suggesting a crucial role in enhancing cognitive development. These results underscore the importance of maternal diet, particularly the consumption of nuts and large fatty fish, in supporting long-term optimal cognitive development in offspring.

## Author contributions

The authors’ responsibilities were as follows – AP-M, JJ, MF: designed research; JJ: coordinated and supervised data collection; AP-M: performed data analyses and interpretation, and drafting and writing of the manuscript, with the support and revision of NA-A, JJ, MF; MV, MG: the principal investigators of the INMA-Sabadell cohort and INMA consortium, respectively, oversaw the data collection, and acquired the funding; JJ: also acquired more funding; and all authors: reviewed and commented on versions of the manuscript, and read and approved the final manuscript.

## Data availability

Data described in the manuscript, code book, and analytic code will be made available upon request. Further inquiries can be directed to the corresponding author.

## Funding

This study was supported by Instituto de Salud Carlos III through the projects “CP14/00108, PI16/00261, and PI21/00266” (cofunded by the European Regional Development Fund “A way to make Europe”). The California Walnut Commission gave funding support. This study was also funded by other grants from Instituto de Salud Carlos III (Red INMA G03/176; CB06/02/0041; PI041436; PI081151 incl. FEDER funds; PI12/01890 incl. FEDER funds; CP13/00054 incl. FEDER funds; PI15/00118 incl. FEDER funds; CP16/00128 incl. FEDER funds; PI16/00118 incl. FEDER funds; PI16/00261 incl. FEDER funds; PI17/01194 incl. FEDER funds; PI17/01340 incl. FEDER funds; PI18/00547 incl. FEDER funds; PI20/01695 incl. FEDER funds), CIBERESP, Generalitat de Catalunya-CIRIT 1999SGR 00241, Generalitat de Catalunya-AGAUR (2009 SGR 501, 2014 SGR 822), Fundació La marató de TV3 (090430), Spanish Ministry of Economy and Competitiveness (SAF2012-32991 incl. FEDER funds), Agence Nationale de Securite Sanitaire de l’Alimentation de l’Environnement et du Travail (1262C0010; EST-2016 RF-21; EST-19 RF-04; 2019/1/233), EU Commission (261357, 308333, 603794; 634453; 825712 and 874583), FIS-PI042018 incl. FEDER funds, FIS-PI09/02311 incl. FEDER funds, FIS-PI13/02429 incl. FEDER funds, FIS-PI18/00909 incl. FEDER funds, CIBERESP, Obra Social Cajastur/Fundación Liberbank and UNIVERSIDAD DE OVIEDO, Department of Health of the Basque Government (2005111093), Provincial Government of Gipuzkoa (DFG06/002), and annual agreements with the municipalities of the study area (Zumarraga, Urretxu , Legazpi, Azkoitia y Azpeitia y Beasain). This study was also funded by grants from UE (FP7-ENV-2011 cod 282957, HEALTH.2010.2.4.5-1, cod 874583, and cod 101136566), Spain: ISCIII (G03/176; FIS-FEDER: PI03/1615, PI04/1509, PI06/1213, PI11/01007, PI11/02591, PI11/02038, PI12/00610, PI13/1944, PI13/2032, PI14/00891, PI14/01687, PI16/1288, PI17/00663, PI19/1338; P 23/1578), Miguel Servet-FEDER CP11/00178, CP15/00025, MSII16/00051, MS20/0006), Spanish Ministry of Universities (Margarita Salas Grant MS21-133, grant CAS21/00008), Generalitat Valenciana (CIAICO/2021/132, BEST/2020/059, AICO 2020/285, AICO/2021/182 and CIDEGENT/2019/064), Consejo General de Enfermería (PNI22_CGE45), FISABIO (UGP 15-230, UGP-15-244, UGP-15-249), and Alicia Koplowitz Foundation 2017. AP-M holds a predoctoral research training (PFIS) contract (grant FI22/00119) awarded by the Instituto de Salud Carlos III. JJ held a Miguel Servet-II contract since May 2023 (grant CPII19/00015) awarded by the Instituto de Salud Carlos III (cofounded by the European Social Fund “Investing in your future”). DH is supported by the LongITools project which has received funding from the European Union's Horizon 2020 research and innovation programme under grant agreement No 874739. The funders have no role in the study design, collection, management, analysis and interpretation of data, writing of the report or decision to submit it for publication.

## Conflict of interest

AP-M reports financial support was provided by Carlos III Health Institute. JJ reports financial support was provided by Carlos III Health Institute. DH reports financial support was provided by European Union Horizon 2020 research and innovation programme. All other authors report no conflicts of interest.
