# Supplementary material for: Maternal prenatal nut and seafood consumption and child neuropsychological function from 4 to 15 years of age: a population-based cohort study
Source: Am J Clin Nutr. 2025 May 5;122(1):274–84. doi: 10.1016/j.ajcnut.2025.04.032 (PMC12308078; doi:10.1016/j.ajcnut.2025.04.032)
Supplement: multimedia component 1 [file mmc1.docx]

**Supplementary Material**

**Supplementary Methods**

### ***Co-variable information***

Trained interviewers administered questionnaires to parents twice during pregnancy (12 and 32 weeks) and at different ages of their children (5, 8, 11, and 14 years) in order to obtain sociodemographic and lifestyle information. We obtained information on maternal age, country of birth, maternal education (primary school or less, secondary, and university) and occupational social class ((I/II: managers/technicians, III: non-manual and IV/V: manual) (1), pre-pregnancy body mass index (BMI, kg/m^2^), omega-3 supplementation during the 1^st^ trimester of pregnancy, and smoking and alcohol consumption during pregnancy (yes/no). Further, maternal verbal intelligence quotient (IQ) was assessed through the Similarities subtest of the Wechsler Adult Intelligence, 3rd edition (WAIS-III) as a proxy at the 5-year visit (2). Total energy intake and relative Mediterranean diet score (rMed) were estimated from the Food Frequency Questionnaire (FFQ). The rMed was constructed using the consumption of fish (without canned and processed fish), vegetables, fruits and nuts, legumes, cereals, meat, dairy products and olive oil. Alcohol consumption was excluded for the calculation of rMed to adapt to pregnant population (3,4). For analyses, the original rMed score was modified without fish (when the exposure was fish intake) and nuts (when the exposure was nuts intake). A similar FFQ of 105 items validated for children at 4-5 years of age, and a shorter 46-item FFQ validated for children aged 7-9 years, were used to gather information on children’s diet in order to estimate seafood and nuts consumption (5,6). Data on breastfeeding (weeks of any breastfeeding) was collected at 6 months and 1 year through a questionnaire. Additionally, trained midwives recorded the child's sex and birth weight during delivery. Finally, child’s age at every visit was calculated based on birth date and the date when the tests were completed.

Mercury (Hg) levels were measured in cord blood (n=1872). Hg was log-transformed to attain normality assumptions of the models. The analytical process has been described elsewhere (7). Organic compounds (OCs), including polychlorinated biphenyls (PCBs, congeners 118, 138, 153 and 180), hexachlorobenzene (HCB), dichlorodiphenyltrichloroethane (DDT) and dichlorodiphenyl dichloroethylene (DDE, a metabolite of DDT), were measured in maternal serum (n=1886) extracted from peripheral veins between the 7th and 26th weeks of pregnancy (median=12.9 weeks). The analytical process has been described elsewhere (8).

**References**

1. Domingo-Salvany A, Bacigalupe A, Carrasco JM, Espelt A, Ferrando J, Borrell C. Propuestas de clase social neoweberiana y neomarxista a partir de la Clasificación Nacional de Ocupaciones 2011. Gac Sanit [Internet] Sociedad Española de Salud Pública y Administración Sanitaria (SESPAS); 2013 [cited 2022 Jan 13];27:263–72. Available from: https://scielo.isciii.es/scielo.php?script=sci_arttext&pid=S0213-91112013000300013&lng=es&nrm=iso&tlng=es

2. Wechsler D. Adult Intelligence Scale-III (Escala de inteligencia de Wechsler para adultos-III) (WAIS-III). TEA Ediciones. Madrid; 2001.

3. Buckland G, González CA, Agudo A, Vilardell M, Berenguer A, Amiano P, Ardanaz E, Arriola L, Barricarte A, Basterretxea M, et al. Adherence to the Mediterranean diet and risk of coronary heart disease in the Spanish EPIC Cohort Study. Am J Epidemiol [Internet] Am J Epidemiol; 2009 [cited 2024 Oct 30];170:1518–29. Available from: https://pubmed.ncbi.nlm.nih.gov/19903723/

4. Fernández-Barrés S, Vrijheid M, Manzano-Salgado CB, Valvi D, Martínez D, Iñiguez C, Jimenez-Zabala A, Riaño-Galán I, Navarrete-Muñoz EM, Santa-Marina L, et al. The Association of Mediterranean Diet during Pregnancy with Longitudinal Body Mass Index Trajectories and Cardiometabolic Risk in Early Childhood. J Pediatr [Internet] J Pediatr; 2019 [cited 2024 Oct 30];206:119-127.e6. Available from: https://pubmed.ncbi.nlm.nih.gov/30429079/

5. Vioque J, Gimenez-Monzo D, Navarrete-Muñoz EM, Garcia-De-la-hera M, Gonzalez-Palacios S, Rebagliato M, Ballester F, Murcia M, Iñiguez C, Granado F. Reproducibility and Validity of a Food Frequency Questionnaire Designed to Assess Diet in Children Aged 4-5 Years. PLoS One [Internet] PLoS One; 2016 [cited 2024 Apr 29];11. Available from: https://pubmed.ncbi.nlm.nih.gov/27898731/

6. Vioque J, Garcia-De-La-Hera M, Gonzalez-Palacios S, Torres-Collado L, Notario-Barandiaran L, Oncina-Canovas A, Soler-Blasco R, Lozano M, Beneito A, Navarrete-Muñoz EM. Reproducibility and Validity of a Short Food Frequency Questionnaire for Dietary Assessment in Children Aged 7^−^9 Years in Spain. Nutrients [Internet] Nutrients; 2019 [cited 2024 Apr 29];11. Available from: https://pubmed.ncbi.nlm.nih.gov/31027207/

7. Ramon R, Murcia M, Aguinagalde X, Amurrio A, Llop S, Ibarluzea J, Lertxundi A, Alvarez-Pedrerol M, Casas M, Vioque J, et al. Prenatal mercury exposure in a multicenter cohort study in Spain. Environ Int Pergamon; 2011;37:597–604.

8. Forns J, Lertxundi N, Aranbarri A, Murcia M, Gascon M, Martinez D, Grellier J, Lertxundi A, Julvez J, Fano E, et al. Prenatal exposure to organochlorine compounds and neuropsychological development up to two years of life. Environ Int Pergamon; 2012;45:72–7.

**Supplementary Figure 1.** Directed Acyclic Graph (DAG) for investigating casual paths (possible confounders) between nut and fish consumption during pregnancy and cognitive performance in the offspring^1^.

**
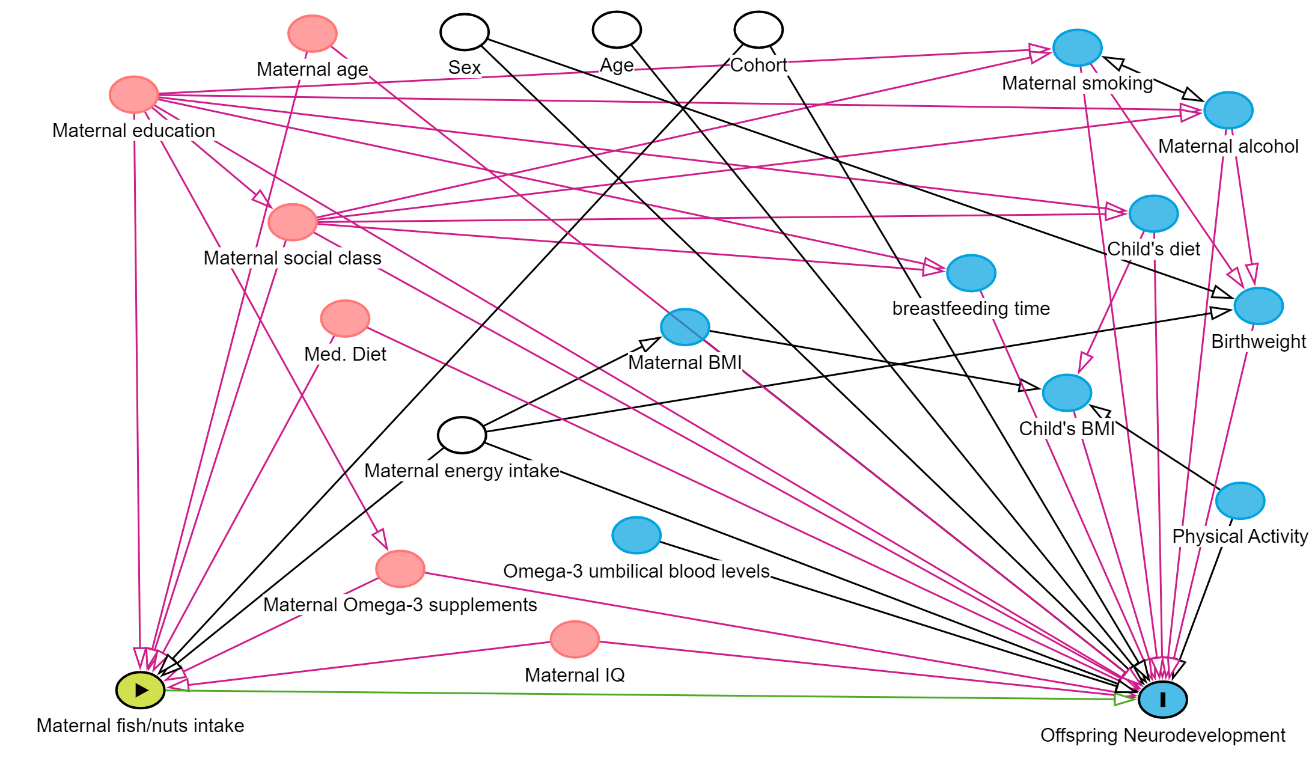
**

^1^ Minimal sufficient adjustment sets containing mother’s energy intake (calories, kcal), child’s sex and children’s age at each visit (years) for estimating the total effect of Maternal fish/nuts intake on Offspring Neurodevelopment: mother’s age at delivery (years), omega-3 supplementation (yes/no), modified rMed score (in tertiles), maternal verbal IQ (continuous, standardized as mean=10, SD=3), maternal education (primary school or less, secondary, university) and occupational social class (I/II: managers/technicians, III: non-manual and IV/V: manual). Cohort (Sabadell, Valencia, Asturias, Gipuzkoa) was used as random effects.

**Supplementary Table 1.** Correlation between umbilical cord blood proportions of C18:3n-3 (ALA), C22:6n-3 (DHA), C20:5n-3 (EPA) and mercury (Hg) according to self-reported nut and fish consumption during the first trimester of pregnancy^1^.

| **Biomarkers^2^** | **Self-reported nut consumption** | | | ***Spearman’s Rho*** | ***p*-value** |
| --- | --- | --- | --- | --- | --- |
|  | **T1** | **T2** | **T3** |  |  |
| C18:3n-3 | 0.11 (0.12) | 0.10 (0.09) | 0.09 (0.09) | -0.0976 | 0.0026 |
| C22:6n-3 | 4.86 (1.52) | 5.01 (1.62) | 5.46 (1.78) | 0.1677 | <0.0001 |
| C20:5n-3 | 0.22 (0.19) | 0.22 (0.14) | 0.27 (0.21) | 0.1295 | 0.0001 |
| DHA+EPA+ALA | 5.19 (1.59) | 5.33 (1.65) | 5.82 (1.82) | 0.1708 | <0.0001 |
|  | **Self-reported fish consumption** | | | ***r*** | ***p*-value** |
|  | **T1** | **T2** | **T3** |  |  |
| C18:3n-3 | 0.11 (0.11) | 0.10 (0.11) | 0.09 (0.07) | -0.0876 | 0.0070 |
| C22:6n-3 | 4.87 (1.56) | 5.16 (1.65) | 5.28 (1.73) | 0.1167 | 0.0003 |
| C20:5n-3 | 0.21 (0.15) | 0.24 (0.19) | 0.25 (0.19) | 0.1197 | 0.0002 |
| DHA+EPA+ALA | 5.19 (1.58) | 5.50 (1.72) | 5.63 (1.78) | 0.1205 | 0.0002 |
| Hg | 0.80 (0.34) | 0.95 (0.32) | 0.99 (0.33) | 0.2581 | <0.0001 |
|  | **Self-reported large fatty fish consumption** | | | ***r*** | ***p*-value** |
|  | **T1** | **T2** | **T3** |  |  |
| C18:3n-3 | 0.10 (0.09) | 0.11 (0.13) | 0.10 (0.10) | -0.0472 | 0.1465 |
| C22:6n-3 | 4.81 (1.50) | 5.46 (1.80) | 5.33 (1.71) | 0.1500 | <0.0001 |
| C20:5n-3 | 0.21 (0.15) | 0.25 (0.17) | 0.26 (0.22) | 0.1676 | <0.0001 |
| DHA+EPA+ALA | 5.12 (1.54) | 5.82 (1.84) | 5.69 (1.77) | 0.1615 | <0.0001 |
| Hg | 0.81 (0.34) | 0.94 (0.30) | 1.03 (0.33) | 0.2750 | <0.0001 |

ALA, Alpha-linolenic acid; DHA, Docosahexaenoic acid; EPA, Eicosapentaenoic acid.

^1^ Mercury is only analyzed to determine its correlation with fish consumption.

^2^ Data are expressed as mean (standard deviation).

**Supplementary Table 2.** Baseline characteristics of the study participants, shown by tertiles of maternal fish consumption in the first trimester of pregnancy.

|  |  | **Tertiles of maternal fish intake** | | |
| --- | --- | --- | --- | --- |
|  | **Total**  N = 2585 | **Low**  n = 861^2^ | **Medium**  n = 862^2^ | **High**  n = 862^2^ |
| Maternal characteristics^1^ |  |  |  |  |
| Fish intake in g/week, median (IQR) | 445.70  (299.72; 630.81) | 249.48  (168.77; 299.50) | 445.66  (400.45; 499.65) | 727.12  (630.81; 884.78) |
| Large fatty fish intake in g/week, median (IQR) | 19.80 (0; 46.93) | 0 (0; 0) | 24.36  (20.99; 29.20) | 56.98  (46.94; 124.28) |
| Age in years, mean (SD) | 30.59 (4.37) | 29.77 (4.69) | 30.72 (4.21) | 31.29 (4.07) |
| Cohort location |  |  |  |  |
| Sabadell | 654 (25.30) | 211 (24.51) | 219 (25.41) | 224 (25.99) |
| Asturias | 482 (18.65) | 128 (14.87) | 142 (16.47) | 212 (24.59) |
| Valencia | 822 (31.80) | 369 (42.86) | 265 (30.74) | 188 (21.81) |
| Gipuzkoa | 627 (24.26) | 153 (17.77) | 236 (27.38) | 238 (27.61) |
| Pre-pregnancy BMI (in kg/m^2^) | 23.58 (4.32) | 23.39 (4.28) | 23.52 (4.22) | 23.82 (4.45) |
| IQ, mean (SD) | 9.94 (2.97) | 9.82 (3.17) | 10.09 (2.94) | 9.92 (2.80) |
| Education |  |  |  |  |
| Primary school or less | 648 (25.11) | 286 (33.26) | 194 (22.56) | 168 (19.51) |
| Secondary school | 1066 (41.30) | 340 (39.53) | 343 (39.88) | 383 (44.48) |
| University or more | 867 (33.59) | 234 (27.21) | 323 (37.56) | 310 (36.00) |
| Country of birth |  |  |  |  |
| Spain | 2364 (91.56) | 747 (86.86) | 795 (92.33) | 822 (95.47) |
| Other | 218 (8.44) | 113 (13.14) | 66 (7.67) | 39 (4.53) |
| Smoking during pregnancy | 1338 (54.97) | 455 (56.38) | 429 (53.42) | 454 (55.10) |
| Alcohol consumption during pregnancy | 789 (30.52) | 234 (27.18) | 276 (32.02) | 279 (32.37) |
| rMED |  |  |  |  |
| Low | 1085 (41.97) | 538 (62.49) | 350 (40.60) | 197 (22.85) |
| Medium | 748 (28.94) | 209 (24.27) | 263 (30.51) | 276 (32.02) |
| High | 752 (29.09) | 114 (13.24) | 249 (28.89) | 389 (45.13) |
| Energy intake in kcals/day, mean (SD) | 2120.01 (556.64) | 2174.65 (586.95) | 2136.37 (574.79) | 2049.06 (496.56) |
| Omega-3 supplementation | 131 (5.07) | 38 (4.41) | 52 (6.03) | 41 (4.76) |
| Child characteristics^1^ |  |  |  |  |
| Sex, female | 1202 (48.43) | 405 (49.15) | 402 (48.96) | 395 (47.19) |
| Birthweight in g |  |  |  |  |
| <3000 | 642 (24.84) | 219 (25.44) | 211 (24.48) | 212 (24.59) |
| 3000-3500 | 1133 (43.83) | 374 (43.44) | 376 (43.62) | 383 (44.43) |
| >3500 | 810 (31.33) | 268 (31.13) | 275 (31.90) | 267 (30.97) |
| Breastfeeding time |  |  |  |  |
| None | 340 (13.15) | 114 (13.24) | 111 (12.88) | 115 (13.34) |
| 0-16 weeks | 582 (22.51) | 220 (25.55) | 186 (21.58) | 176 (20.42) |
| 16-24 weeks | 363 (14.04) | 116 (13.47) | 118 (13.69) | 129 (14.97) |
| > 24 weeks | 1300 (50.29) | 411 (47.74) | 447 (51.86) | 442 (51.28) |
| Mercury cord-blood levels, mean (SD)^3^ | 0.91 (0.34) | 0.80 (0.34) | 0.95 (0.32) | 0.99 (0.33) |
| Omega-3 fatty acids umbilical cord blood levels^4^ |  |  |  |  |
| ALA in %, mean (SD) | 0.10 (0.10) | 0.11 (0.11) | 0.10 (0.11) | 0.09 (0.09) |
| DHA in %, mean (SD) | 5.11 (1.66) | 4.87 (1.56) | 5.16 (1.65) | 5.28 (1.73) |
| EPA in %, mean (SD) | 0.23 (0.18) | 0.21 (0.15) | 0.24 (0.19) | 0.25 (0.19) |

ALA, Alpha-linolenic acid; BMI, Body mass index; DHA, Docosahexaenoic acid; EPA, Eicosapentaenoic acid; IQ, Intelligence quotient; IQR, Interquartile range; rMed, relative Mediterranean diet score; SD, Standard deviation.

^1^ Unless otherwise indicated, data are expressed as number (percentage) of participants. Percentages have been rounded and may not total 100.

^2^ Some of totals may not match the total number of subjects due to missings.

^3^ Cord-blood mercury (log-transformed) was only measured in a sub-set of participants (N = 1872), n = 602 for the first tertile, n = 630 for the second tertile, and n = 640 for the third tertile.

^4^ Omega-3 fatty acids were only measured in a sub-set of participants (N = 948), n = 302 for the first tertile, n = 315 for the second tertile, and n = 331 for the third tertile of maternal fish intake.

**Supplementary Table 3.** Child neuropsychological scores at each visit according to maternal nut consumption during the first trimester of pregnancy.

| **Neuropsychological outcome**^1, 2^ |  |  | **Tertiles of maternal nut intake** | | | ***p* values^3^** |
| --- | --- | --- | --- | --- | --- | --- |
|  |  | **N** | **Low** | **Medium** | **High** |  |
|  | Year visit |  |  |  |  |  |
| Attention^4^  ANT  (HRT-SE) | 5 years^6^ | 1331 | 0.03 (0.98) | -0.02 (0.96) | -0.06 (1.07) | 0.062 |
|  | 7-8 years^6^ | 1655 | 0.02 (1.01) | 0.08 (1.00) | -0.18 (0.99) | 0.885 |
|  | 11 years^6^ | 1386 | -0.03 (0.96) | 0.04 (1.01) | -0.09 (0.99) | 0.592 |
| (HRT-mean) | 5 years | 1331 | -0.01 (1.00) | 0.01 (1.01) | -0.03 (0.99) | 0.962 |
|  | 7-8 years | 1655 | -0.04 (0.98) | 0.05 (1.02) | -0.11 (0.95) | 0.234 |
|  | 11 years | 1386 | 0.01 (1.04) | 0.07 (1.02) | -0.12 (0.94) | 0.081 |
| Working Memory^5^  N-back  (d2’) | 7-8 years | 1599 | -0.01 (0.96) | -0.05 (1.02) | 0.12 (1.01) | 0.336 |
|  | 11 years | 1378 | 0.03 (0.97) | -0.09 (0.94) | 0.08 (0.94) | 0.019 |
|  | 15 years^7^ | 811 | -0.08 (0.99) | 0.02 (1.03) | 0.06 (0.94) | 0.406 |
| (d3’) | 7-8 years | 1550 | -0.03 (1.02) | -0.04 (1.04) | 0.12 (0.95) | 0.122 |
|  | 11 years | 1359 | -0.03 (0.98) | -0.03 (1.09) | 0.08 (0.92) | 0.001 |
|  | 15 years | 812 | -0.09 (1.06) | -0.03 (0.96) | 0.15 (0.94) | 0.098 |
| Fluid Intelligence^5^  (PMA-R & Raven) | 7-9 years^7^ | 1181 | -0.00 (1.00) | 0.04 (0.97) | -0.00 (1.02) | 0.665 |
|  | 11-15 years^8^ | 735 | -0.08 (1.03) | -0.08 (0.98) | 0.13 (0.97) | 0.544 |

ANT, Attention Network Test; HRT-SE, Hit Reaction Time - Standard Error; PMA-R, Primary Mental Abilities – Reasoning.

^1^ Neuropsychological outcome scores were standardized as z-scores.

^2^ Data is expressed as mean (SD).

^3^ p-values calculated using one-way ANOVA.

^4^ Lower scores indicate better performance.

^5^ Higher scores indicate better performance.

^6^ All cohorts were involved (Gipuzkoa, Valencia, Asturias and Sabadell).

^7^ All cohorts were involved except Asturias (Gipuzkoa, Valencia and Sabadell).

^8^ All cohorts were involved except Sabadell (Gipuzkoa, Valencia and Asturias).

**Supplementary Table 4.** Child neuropsychological scores at each visit according to maternal large fatty fish consumption during the first trimester of pregnancy.

| **Neuropsychological outcome**^1, 2^ |  |  | **Tertiles of maternal large fatty fish intake** | | | ***p* values**^3^ |
| --- | --- | --- | --- | --- | --- | --- |
|  |  | **N** | **Low** | **Medium** | **High** |  |
|  | Year visit |  |  |  |  |  |
| Attention^4^  ANT  (HRT-SE) | 5 years^6^ | 1331 | 0.04 (1.09) | -0.11 (0.84) | -0.04 (0.98) | < 0.001 |
|  | 7-8 years^6^ | 1655 | 0.10 (0.99) | -0.02 (0.96) | -0.98 (1.02) | 0.394 |
|  | 11 years^6^ | 1386 | 0.07 (1.02) | -0.13 (0.91) | -0.09 (0.99) | 0.082 |
| (HRT-mean) | 5 years | 1331 | -0.02 (0.99) | -0.10 (0.88) | 0.06 (1.07) | 0.001 |
|  | 7-8 years | 1655 | 0.06 (0.99) | 0.01 (0.99) | -0.18 (0.97) | 0.801 |
|  | 11 years | 1386 | 0.06 (1.04) | -0.11 (0.93) | -0.06 (1.00) | 0.071 |
| Working Memory^5^  N-back  (d2’) | 7-8 years | 1599 | -0.05 (0.98) | -0.02 (1.01) | 0.13 (1.00) | 0.817 |
|  | 11 years | 1378 | -0.06 (1.03) | 0.00 (1.01) | 0.10 (0.93) | 0.041 |
|  | 15 years^7^ | 811 | -0.02 (1.03) | 0.06 (0.98) | 0.01 (0.93) | 0.260 |
| (d3’) | 7-8 years | 1550 | -0.09 (1.03) | 0.05 (0.97) | 0.13 (0.98) | 0.376 |
|  | 11 years | 1359 | -0.05 (0.99) | 0.03 (0.99) | 0.08 (1.01) | 0.820 |
|  | 15 years | 812 | 0.03 (0.97) | 0.08 (1.01) | -0.07 (1.00) | 0.794 |
| Fluid Intelligence^5^  (PMA-R & Raven) | 7-9 years^7^ | 1181 | -0.08 (1.03) | 0.07 (0.94) | 0.10 (0.98) | 0.257 |
|  | 11-15 years^8^ | 735 | -0.01 (0.99) | 0.15 (1.02) | -0.06 (0.99) | 0.883 |

ANT, Attention Network Test; HRT-SE, Hit Reaction Time - Standard Error; PMA-R, Primary Mental Abilities – Reasoning.

^1^ Neuropsychological outcome scores were standardized as z-scores.

^2^ Data is expressed as mean (SD).

^3^ p-values calculated using one-way ANOVA.

^4^ Lower scores indicate better performance.

^5^ Higher scores indicate better performance.

^6^ All cohorts were involved (Gipuzkoa, Valencia, Asturias and Sabadell).

^7^ All cohorts were involved except Asturias (Gipuzkoa, Valencia and Sabadell).

^8^ All cohorts were involved except Sabadell (Gipuzkoa, Valencia and Asturias).

**Supplementary Table 5.** Association between nut consumption during pregnancy and offspring neuropsychological function up to 15 years of age, adjusted by post-natal nut intake (mean at 4 and 8 years old).

| **Neuropsychological outcome**^1^ |  | **Fully adjusted**^2^ | | | **Fully adjusted + post-natal nut intake**^3^ | | |
| --- | --- | --- | --- | --- | --- | --- | --- |
|  |  | **β** | **95% CI** | ***p*-value** | **β** | **95% CI** | ***p*-value** |
|  | Maternal nut intake in the 1^st^ trimester |  |  |  |  |  |  |
| Attention^4^  ANT  (HRT-SE)  N = 1458 |  |  |  |  |  |  |  |
|  | Lowest tertile | Ref. | | | | | |
|  | Middle tertile | 0.02 | -0.07; 0.12 | 0.620 | 0.03 | -0.07; 0.13 | 0.531 |
|  | Higher tertile | -0.10 | -0.20; -0.01 | 0.028 | -0.09 | -0.18; 0.00 | 0.060 |
|  | Tertiles in continuous | -0.05 | -0.10; -0.01 | 0.028 | -0.04 | -0.09; 0.00 | 0.059 |
| (HRT mean) | Lowest tertile | Ref. | | | | | |
| N = 1458 | Middle tertile | 0.04 | -0.06; 0.13 | 0.424 | 0.04 | -0.05; 0.14 | 0.350 |
|  | Higher tertile | -0.05 | -0.14; 0.04 | 0.299 | -0.03 | -0.13; 0.06 | 0.470 |
|  | Tertiles in continuous | -0.02 | -0.07; 0.02 | 0.990 | -0.02 | -0.06; 0.03 | 0.462 |
| Working memory^5^  N-back  (d2’)  N = 1444 |  |  |  |  |  |  |  |
|  | Lowest tertile | Ref. | | | | | |
|  | Middle tertile | 0.01 | -0.08; 0.11 | 0.784 | 0.00 | -0.09; 0.10 | 0.921 |
|  | Higher tertile | 0.08 | -0.01; 0.17 | 0.092 | 0.06 | -0.03; 0.15 | 0.186 |
|  | Tertiles in continuous | 0.04 | -0.01; 0.08 | 0.091 | 0.03 | -0.01; 0.08 | 0.183 |
| (d3’)  N = 1436 | Lowest tertile | Ref. | | | | | |
|  | Middle tertile | 0.06 | -0.04: 0.15 | 0.225 | 0.06 | -0.04; 0.15 | 0.251 |
|  | Higher tertile | 0.13 | 0.03; 0.22 | 0.008 | 0.12 | 0.03; 0.22 | 0.012 |
|  | Tertiles in continuous | 0.06 | 0.02; 0.11 | 0.008 | 0.07 | 0.01; 0.11 | 0.012 |
| Fluid intelligence^5^  (PMA-R & Raven)  N = 1192 | Lowest tertile | Ref. | | | | | |
|  | Middle tertile | -0.05 | -0.17; 0.08 | 0.471 | -0.05 | -0.17; 0.08 | 0.461 |
|  | Higher tertile | 0.09 | -0.03; 0.21 | 0.151 | 0.09 | -0.04; 0.21 | 0.168 |
|  | Tertiles in continuous | 0.05 | -0.02; 0.11 | 0.144 | 0.04 | -0.02; 0.10 | 0.159 |

ANT, Attention Network Test; CI, Confidence Interval; HRT-SE, Hit Reaction Time - Standard Error; PMA-R, Primary Mental Abilities – Reasoning.

^1^ Neuropsychological outcome scores were standardized as z-scores.

^2^ Data was calculated using a linear mixed model with cohort and individuals as random effects, visit as random slope, and adjusted for mother’s age at delivery, children’s sex and age on each visit, mother’s energy intake, maternal omega-3 supplementation during pregnancy, maternal IQ, rMed score (without nuts), maternal education and maternal social class. The N of the sample was matched to the N of the sensitivity analysis model.

^3^ Fully adjusted model + post-natal nut intake. Post-natal nut intake was calculated as a mean of nut consumption at 4 and 8 years of age.

^4^ Lower scores indicate better performance.

^5^ Higher scores indicate better performance.

**Supplementary Table 6.** Association between nut consumption during pregnancy and offspring neuropsychological function up to 15 years of age, adjusted by breastfeeding duration.

| **Neuropsychological outcome**^1^ |  | **Fully adjusted**^2^ | | | **Fully adjusted + breastfeeding duration**^3^ | | |
| --- | --- | --- | --- | --- | --- | --- | --- |
|  |  | **β** | **95% CI** | ***p*-value** | **β** | **95% CI** | ***p*-value** |
|  | Maternal nut intake in the 1^st^ trimester |  |  |  |  |  |  |
| Attention^4^  ANT  (HRT-SE)  N = 1661 |  |  |  |  |  |  |  |
|  | Lowest tertile | Ref. | | | | | |
|  | Middle tertile | 0.05 | -0.04; 0.14 | 0.274 | 0.05 | -0.04; 0.14 | 0.271 |
|  | Higher tertile | -0.10 | -0.19; -0.01 | 0.031 | -0.10 | -0.19; -0.01 | 0.033 |
|  | Tertiles in continuous | -0.05 | -0.09; -0.00 | 0.033 | -0.05 | -0.09; -0.00 | 0.035 |
| (HRT mean) | Lowest tertile | Ref. | | | | | |
| N = 1661 | Middle tertile | 0.07 | -0.02; 0.16 | 0.112 | 0.07 | -0.02; 0.16 | 0.113 |
|  | Higher tertile | -0.05 | -0.14; 0.03 | 0.216 | -0.05 | -0.14; 0.03 | 0.211 |
|  | Tertiles in continuous | -0.03 | -0.07; 0.02 | 0.220 | -0.03 | -0.07; 0.02 | 0.216 |
| Working memory^5^  N-back  (d2’)  N = 1538 |  |  |  |  |  |  |  |
|  | Lowest tertile | Ref. | | | | | |
|  | Middle tertile | -0.02 | -0.11; 0.08 | 0.746 | -0.01 | -0.11; 0.08 | 0.752 |
|  | Higher tertile | 0.09 | -0.00; 0.18 | 0.054 | 0.09 | -0.00; 0.18 | 0.051 |
|  | Tertiles in continuous | 0.04 | -0.00; 0.09 | 0.052 | 0.04 | 0.00; 0.09 | 0.050 |
| (d3’)  N = 1530 | Lowest tertile | Ref. | | | | | |
|  | Middle tertile | 0.05 | -0.04: 0.15 | 0.285 | 0.05 | -0.04: 0.15 | 0.280 |
|  | Higher tertile | 0.12 | 0.03; 0.22 | 0.007 | 0.13 | 0.04; 0.22 | 0.006 |
|  | Tertiles in continuous | 0.06 | 0.02; 0.11 | 0.007 | 0.06 | 0.02; 0.11 | 0.006 |
| Fluid intelligence^5^  (PMA-R & Raven)  N = 1273 | Lowest tertile | Ref. | | | | | |
|  | Middle tertile | -0.04 | -0.16; 0.08 | 0.541 | -0.04 | -0.16; 0.08 | 0.533 |
|  | Higher tertile | 0.10 | -0.01; 0.22 | 0.082 | 0.10 | -0.02; 0.22 | 0.088 |
|  | Tertiles in continuous | 0.05 | -0.01; 0.11 | 0.079 | 0.05 | -0.01; 0.11 | 0.084 |

ANT, Attention Network Test; CI, Confidence Interval; HRT-SE, Hit Reaction Time - Standard Error; PMA-R, Primary Mental Abilities – Reasoning.

^1^ Neuropsychological outcome scores were standardized as z-scores.

^2^ Data was calculated using a linear mixed model with cohort and individuals as random effects, visit as random slope, and adjusted for mother’s age at delivery, children’s sex and age on each visit, mother’s energy intake, maternal omega-3 supplementation during pregnancy, maternal IQ, rMed score (without nuts), maternal education and maternal social class. The N of the sample was matched to the N of the sensitivity analysis model.

^3^ Fully adjusted model + breastfeeding duration (in weeks).

^4^ Lower scores indicate better performance.

^5^ Higher scores indicate better performance.

**Supplementary Table 7.** Association between large fatty fish consumption during pregnancy and offspring neuropsychological function up to 15 years of age, adjusted by post-natal fish intake (mean at 4 and 8 years old).

| **Neuropsychological outcome**^1^ |  | **Fully adjusted**^2^ | | | **Fully adjusted + post-natal fish intake**^3^ | | |
| --- | --- | --- | --- | --- | --- | --- | --- |
|  |  | **β** | **95% CI** | ***p*-value** | **β** | **95% CI** | ***p*-value** |
|  | Maternal fish intake in the 1^st^ trimester |  |  |  |  |  |  |
| Attention^4^  ANT  (HRT-SE)  N = 1458 |  |  |  |  |  |  |  |
|  | Lowest tertile | Ref. | | | | | |
|  | Middle tertile | -0.11 | -0.21; -0.01 | 0.036 | -0.11 | -0.21; -0.01 | 0.031 |
|  | Higher tertile | -0.09 | -0.18; -0.00 | 0.044 | -0.10 | -0.18; -0.01 | 0.035 |
|  | Tertiles in continuous | -0.05 | -0.09; -0.00 | 0.036 | -0.05 | -0.09; -0.01 | 0.028 |
| (HRT mean) | Lowest tertile | Ref. | | | | | |
| N = 1458 | Middle tertile | -0.08 | -0.18; 0.01 | 0.097 | -0.08 | -0.18; 0.02 | 0.105 |
|  | Higher tertile | -0.06 | -0.15; 0.02 | 0.151 | -0.06 | -0.14; 0.03 | 0.179 |
|  | Tertiles in continuous | -0.03 | -0.08; 0.01 | 0.119 | -0.03 | -0.07; 0.01 | 0.143 |
| Working Memory^5^  N-back  (d2’)  N = 1444 |  |  |  |  |  |  |  |
|  | Lowest tertile | Ref. | | | | | |
|  | Middle tertile | -0.00 | -0.10; 0.10 | 0.966 | -0.00 | -0.10; 0.10 | 0.957 |
|  | Higher tertile | 0.04 | -0.05; 0.13 | 0.354 | 0.04 | -0.05; 0.12 | 0.407 |
|  | Tertiles in continuous | 0.02 | -0.02; 0.06 | 0.369 | 0.02 | -0.03; 0.06 | 0.422 |
| (d3’)  N = 1436 | Lowest tertile | Ref. | | | | | |
|  | Middle tertile | 0.10 | 0.00; 0.20 | 0.046 | 0.10 | 0.00; 0.20 | 0.049 |
|  | Higher tertile | 0.02 | -0.07; 0.11 | 0.681 | 0.02 | -0.07; 0.11 | 0.690 |
|  | Tertiles in continuous | 0.01 | -0.03; 0.06 | 0.589 | 0.01 | -0.03; 0.06 | 0.601 |
| Fluid Intelligence^5^  (PMA-R & Raven)  N = 1192 | Lowest tertile | Ref. | | | | | |
|  | Middle tertile | 0.18 | 0.06; 0.31 | 0.005 | 0.18 | 0.05; 0.31 | 0.006 |
|  | Higher tertile | 0.16 | 0.05; 0.28 | 0.006 | 0.16 | 0.04; 0.27 | 0.008 |
|  | Tertiles in continuous | 0.08 | 0.03; 0.14 | 0.004 | 0.08 | 0.02; 0.14 | 0.006 |

ANT, Attention Network Test; CI, Confidence Interval; HRT-SE, Hit Reaction Time - Standard Error; PMA-R, Primary Mental Abilities – Reasoning.

^1^ Neuropsychological outcome scores were standardized as z-scores.

^2^ Data was calculated using a linear mixed model with cohort and individuals as random effects, visit as random slope, and adjusted for mother’s age at delivery, children’s sex and age on each visit, mother’s energy intake, maternal omega-3 supplementation during pregnancy, maternal IQ, rMed score (without fish), maternal education and maternal social class. The N of the sample was matched to the N of the sensitivity analysis model.

^3^ Fully adjusted model + post-natal fish intake. Post-natal fish intake was calculated as a mean of fish consumption at 4 and 8 years of age.

^4^ Lower scores indicate better performance.

^5^ Higher scores indicate better performance.

**Supplementary Table 8.** Association between large fatty fish consumption during pregnancy and offspring neuropsychological function up to 15 years of age, adjusted by breastfeeding duration.

| **Neuropsychological outcome**^1^ |  | **Fully adjusted**^2^ | | | **Fully adjusted + breastfeeding duration**^3^ | | |
| --- | --- | --- | --- | --- | --- | --- | --- |
|  |  | **β** | **95% CI** | ***p*-value** | **β** | **95% CI** | ***p*-value** |
|  | Maternal fish intake in the 1^st^ trimester |  |  |  |  |  |  |
| Attention^4^  ANT  (HRT-SE)  N = 1661 |  |  |  |  |  |  |  |
|  | Lowest tertile | Ref. | | | | | |
|  | Middle tertile | -0.13 | -0.23; -0.03 | 0.010 | -0.13 | -0.22; -0.03 | 0.011 |
|  | Higher tertile | -0.11 | -0.20; -0.03 | 0.009 | -0.11 | -0.20; -0.03 | 0.010 |
|  | Tertiles in continuous | -0.06 | -0.10; -0.02 | 0.006 | -0.06 | -0.10; -0.02 | 0.007 |
| (HRT mean) | Lowest tertile | Ref. | | | | | |
| N = 1661 | Middle tertile | -0.09 | -0.18; 0.00 | 0.061 | -0.09 | -0.18; 0.00 | 0.058 |
|  | Higher tertile | -0.08 | -0.16; 0.00 | 0.063 | -0.08 | -0.16; 0.00 | 0.055 |
|  | Tertiles in continuous | -0.04 | -0.08; -0.00 | 0.048 | -0.04 | -0.08; -0.00 | 0.046 |
| Working Memory^5^  N-back  (d2’)  N = 1538 |  |  |  |  |  |  |  |
|  | Lowest tertile | Ref. | | | | | |
|  | Middle tertile | 0.02 | -0.07; 0.12 | 0.646 | 0.02 | -0.07; 0.12 | 0.633 |
|  | Higher tertile | 0.04 | -0.04; 0.13 | 0.341 | 0.04 | -0.04; 0.13 | 0.326 |
|  | Tertiles in continuous | 0.02 | -0.02; 0.06 | 0.338 | 0.02 | -0.02; 0.06 | 0.324 |
| (d3’)  N = 1530 | Lowest tertile | Ref. | | | | | |
|  | Middle tertile | 0.09 | -0.01; 0.19 | 0.073 | 0.09 | -0.01; 0.19 | 0.064 |
|  | Higher tertile | 0.02 | -0.07; 0.11 | 0.645 | 0.03 | -0.06; 0.11 | 0.572 |
|  | Tertiles in continuous | 0.01 | -0.03; 0.06 | 0.559 | 0.02 | -0.03; 0.06 | 0.491 |
| Fluid Intelligence^5^  (PMA-R & Raven)  N = 1273 | Lowest tertile | Ref. | | | | | |
|  | Middle tertile | 0.18 | 0.06; 0.31 | 0.005 | 0.18 | 0.05; 0.30 | 0.005 |
|  | Higher tertile | 0.16 | 0.05; 0.27 | 0.005 | 0.16 | 0.05; 0.27 | 0.006 |
|  | Tertiles in continuous | 0.08 | 0.03; 0.14 | 0.003 | 0.08 | 0.03; 0.14 | 0.004 |

ANT, Attention Network Test; CI, Confidence Interval; HRT-SE, Hit Reaction Time - Standard Error; PMA-R, Primary Mental Abilities – Reasoning.

^1^ Neuropsychological outcome scores were standardized as z-scores.

^2^ Data was calculated using a linear mixed model with cohort and individuals as random effects, visit as random slope, and adjusted for mother’s age at delivery, children’s sex and age on each visit, mother’s energy intake, maternal omega-3 supplementation during pregnancy, maternal IQ, rMed score (without fish), maternal education and maternal social class. The N of the sample was matched to the N of the sensitivity analysis model.

^3^ Fully adjusted model + breastfeeding duration (in weeks).

^4^ Lower scores indicate better performance.

^5^ Higher scores indicate better performance.

**Supplementary Table 9.** Association between large fatty fish consumption during pregnancy and offspring neuropsychological function up to 15 years of age, adjusted by cord-blood mercury levels.

| **Neuropsychological outcome**^1^ |  | **Fully adjusted**^2^ | | | **Fully adjusted + Hg cord-blood**^3^ | | |
| --- | --- | --- | --- | --- | --- | --- | --- |
|  |  | **β** | **95% CI** | ***p*-value** | **β** | **95% CI** | ***p*-value** |
|  | Maternal fish intake in the 1^st^ trimester |  |  |  |  |  |  |
| Attention^4^  ANT  (HRT-SE)  N = 1273 |  |  |  |  |  |  |  |
|  | Lowest tertile | Ref. | | | | | |
|  | Middle tertile | -0.14 | -0.25; -0.02 | 0.017 | -0.13 | -0.25; -0.02 | 0.023 |
|  | Higher tertile | -0.17 | -0.26; -0.07 | 0.001 | -0.16 | -0.26; -0.06 | 0.002 |
|  | Tertiles in continuous | -0.08 | -0.13; -0.04 | 0.001 | -0.08 | -0.13; -0.03 | 0.002 |
| (HRT mean) | Lowest tertile | Ref. | | | | | |
| N = 1273 | Middle tertile | -0.10 | -0.21; 0.01 | 0.069 | -0.10 | -0.21; 0.01 | 0.075 |
|  | Higher tertile | -0.12 | -0.21; -0.03 | 0.009 | -0.12 | -0.21; -0.02 | 0.013 |
|  | Tertiles in continuous | -0.06 | -0.11; -0.02 | 0.008 | -0.06 | -0.11; -0.01 | 0.012 |
| Working Memory^5^  N-back  (d2’)  N = 1179 |  |  |  |  |  |  |  |
|  | Lowest tertile | Ref. | | | | | |
|  | Middle tertile | 0.01 | -0.10; 0.12 | 0.811 | 0.00 | -0.11; 0.11 | 0.950 |
|  | Higher tertile | 0.02 | -0.08; 0.12 | 0.675 | 0.01 | -0.10; 0.11 | 0.889 |
|  | Tertiles in continuous | 0.01 | -0.04; 0.06 | 0.672 | 0.00 | -0.05; 0.05 | 0.889 |
| (d3’)  N = 1175 | Lowest tertile | Ref. | | | | | |
|  | Middle tertile | 0.09 | -0.02; 0.20 | 0.118 | 0.08 | -0.03; 0.19 | 0.166 |
|  | Higher tertile | 0.03 | -0.07; 0.13 | 0.554 | 0.02 | -0.08; 0.12 | 0.743 |
|  | Tertiles in continuous | 0.02 | -0.03; 0.07 | 0.498 | 0.01 | -0.04; 0.06 | 0.692 |
| Fluid Intelligence^5^  (PMA-R & Raven)  N = 995 | Lowest tertile | Ref. | | | | | |
|  | Middle tertile | 0.17 | 0.03; 0.31 | 0.020 | 0.16 | 0.02; 0.30 | 0.029 |
|  | Higher tertile | 0.13 | 0.00; 0.26 | 0.043 | 0.11 | -0.01; 0.24 | 0.081 |
|  | Tertiles in continuous | 0.07 | 0.01; 0.13 | 0.034 | 0.06 | -0.00; 0.12 | 0.067 |

ANT, Attention Network Test; CI, Confidence Interval; Hg, Mercury; HRT-SE, Hit Reaction Time - Standard Error; PMA-R, Primary Mental Abilities – Reasoning.

^1^ Neuropsychological outcome scores were standardized as z-scores.

^2^ Data was calculated using a linear mixed model with cohort and individuals as random effects, visit as random slope, and adjusted for mother’s age at delivery, children’s sex and age on each visit, mother’s energy intake, maternal omega-3 supplementation during pregnancy, maternal IQ, rMed score (without fish), maternal education and maternal social class. The N of the sample was matched to the N of the sensitivity analysis model.

^3^ Fully adjusted model + mercury cord-blood levels

^4^ Lower scores indicate better performance.

^5^ Higher scores indicate better performance.

**Supplementary Table 10.** Association between large fatty fish consumption during pregnancy and offspring neuropsychological function up to 15 years of age, adjusted by maternal hexachlorobenzene (HCB) serum levels at 12 weeks of pregnancy.

| **Neuropsychological outcome**^1^ |  | **Fully adjusted**^2^ | | | **Fully adjusted + HCB**^3^ | | |
| --- | --- | --- | --- | --- | --- | --- | --- |
|  |  | **β** | **95% CI** | ***p*-value** | **β** | **95% CI** | ***p*-value** |
|  | Maternal fish intake in the 1^st^ trimester |  |  |  |  |  |  |
| Attention^4^  ANT  (HRT-SE)  N = 1273 |  |  |  |  |  |  |  |
|  | Lowest tertile | Ref. | | | | | |
|  | Middle tertile | -0.13 | -0.24; -0.02 | 0.021 | -0.13 | -0.24; -0.02 | 0.020 |
|  | Higher tertile | -0.10 | -0.20; -0.01 | 0.030 | -0.11 | -0.20; -0.01 | 0.026 |
|  | Tertiles in continuous | -0.05 | -0.10; -0.01 | 0.023 | -0.06 | -0.10; -0.01 | 0.020 |
| (HRT mean) | Lowest tertile | Ref. | | | | | |
| N = 1273 | Middle tertile | -0.10 | -0.21; 0.01 | 0.062 | -0.10 | -0.21; 0.00 | 0.061 |
|  | Higher tertile | -0.07 | -0.17; 0.02 | 0.109 | -0.08 | -0.17; 0.01 | 0.101 |
|  | Tertiles in continuous | -0.04 | -0.08; 0.01 | 0.094 | -0.04 | -0.09; 0.01 | 0.088 |
| Working Memory^5^  N-back  (d2’)  N = 1179 |  |  |  |  |  |  |  |
|  | Lowest tertile | Ref. | | | | | |
|  | Middle tertile | 0.04 | -0.07; 0.15 | 0.480 | 0.04 | -0.07; 0.15 | 0.478 |
|  | Higher tertile | 0.04 | -0.06; 0.14 | 0.420 | 0.04 | -0.06; 0.14 | 0.411 |
|  | Tertiles in continuous | 0.02 | -0.03; 0.07 | 0.407 | 0.02 | -0.03; 0.07 | 0.398 |
| (d3’)  N = 1175 | Lowest tertile | Ref. | | | | | |
|  | Middle tertile | 0.12 | 0.01; 0.24 | 0.032 | 0.12 | 0.01; 0.24 | 0.033 |
|  | Higher tertile | 0.00 | -0.10; 0.10 | 0.928 | 0.00 | -0.10; 0.10 | 0.954 |
|  | Tertiles in continuous | 0.01 | -0.04; 0.06 | 0.820 | 0.01 | -0.05; 0.06 | 0.845 |
| Fluid Intelligence^5^  (PMA-R & Raven)  N = 995 | Lowest tertile | Ref. | | | | | |
|  | Middle tertile | 0.15 | 0.00; 0.29 | 0.047 | 0.15 | 0.00; 0.29 | 0.047 |
|  | Higher tertile | 0.13 | 0.00; 0.26 | 0.048 | 0.13 | 0.00; 0.26 | 0.045 |
|  | Tertiles in continuous | 0.07 | 0.00; 0.13 | 0.040 | 0.07 | 0.00; 0.13 | 0.038 |

ANT, Attention Network Test; CI, Confidence Interval; HCB, hexachlorobenzene; HRT-SE, Hit Reaction Time - Standard Error; PMA-R, Primary Mental Abilities – Reasoning.

^1^ Neuropsychological outcome scores were standardized as z-scores.

^2^ Data was calculated using a linear mixed model with cohort and individuals as random effects, visit as random slope, and adjusted for mother’s age at delivery, children’s sex and age on each visit, mother’s energy intake, maternal omega-3 supplementation during pregnancy, maternal IQ, rMed score (without fish), maternal education and maternal social class. The N of the sample was matched to the N of the sensitivity analysis model.

^3^ Fully adjusted model + maternal HCB serum levels at 12 weeks of pregnancy.

^4^ Lower scores indicate better performance.

^5^ Higher scores indicate better performance.

**Supplementary Table 11.** Association between large fatty fish consumption during pregnancy and offspring neuropsychological function up to 15 years of age, adjusted by maternal dichlorodiphenyl dichloroethylene (DDE) serum levels at 12 weeks of pregnancy.

| **Neuropsychological outcome**^1^ |  | **Fully adjusted**^2^ | | | **Fully adjusted + DDE**^3^ | | |
| --- | --- | --- | --- | --- | --- | --- | --- |
|  |  | **β** | **95% CI** | ***p*-value** | **β** | **95% CI** | ***p*-value** |
|  | Maternal fish intake in the 1^st^ trimester |  |  |  |  |  |  |
| Attention^4^  ANT  (HRT-SE)  N = 1273 |  |  |  |  |  |  |  |
|  | Lowest tertile | Ref. | | | | | |
|  | Middle tertile | -0.13 | -0.24; -0.02 | 0.021 | -0.13 | -0.24; -0.02 | 0.019 |
|  | Higher tertile | -0.10 | -0.20; -0.01 | 0.030 | -0.10 | -0.20; -0.01 | 0.030 |
|  | Tertiles in continuous | -0.05 | -0.10; -0.01 | 0.023 | -0.05 | -0.10; -0.01 | 0.023 |
| (HRT mean) | Lowest tertile | Ref. | | | | | |
| N = 1273 | Middle tertile | -0.10 | -0.21; 0.01 | 0.062 | -0.10 | -0.21; 0.00 | 0.060 |
|  | Higher tertile | -0.07 | -0.17; 0.02 | 0.109 | -0.07 | -0.17; 0.02 | 0.109 |
|  | Tertiles in continuous | -0.04 | -0.08; 0.01 | 0.094 | -0.04 | -0.08; 0.01 | 0.088 |
| Working Memory^5^  N-back  (d2’)  N = 1179 |  |  |  |  |  |  |  |
|  | Lowest tertile | Ref. | | | | | |
|  | Middle tertile | 0.04 | -0.07; 0.15 | 0.480 | 0.04 | -0.07; 0.15 | 0.463 |
|  | Higher tertile | 0.04 | -0.06; 0.14 | 0.420 | 0.04 | -0.06; 0.14 | 0.418 |
|  | Tertiles in continuous | 0.02 | -0.03; 0.07 | 0.407 | 0.02 | -0.03; 0.07 | 0.404 |
| (d3’)  N = 1175 | Lowest tertile | Ref. | | | | | |
|  | Middle tertile | 0.12 | 0.01; 0.24 | 0.032 | 0.12 | 0.01; 0.24 | 0.033 |
|  | Higher tertile | 0.00 | -0.10; 0.10 | 0.928 | 0.00 | -0.10; 0.10 | 0.930 |
|  | Tertiles in continuous | 0.01 | -0.04; 0.06 | 0.820 | 0.01 | -0.04; 0.06 | 0.824 |
| Fluid Intelligence^5^  (PMA-R & Raven)  N = 995 | Lowest tertile | Ref. | | | | | |
|  | Middle tertile | 0.15 | 0.00; 0.29 | 0.047 | 0.15 | 0.01; 0.29 | 0.042 |
|  | Higher tertile | 0.13 | 0.00; 0.26 | 0.048 | 0.13 | 0.00; 0.26 | 0.048 |
|  | Tertiles in continuous | 0.07 | 0.00; 0.13 | 0.040 | 0.07 | 0.00; 0.13 | 0.040 |

ANT, Attention Network Test; CI, Confidence Interval; DDE, dichlorodiphenyl dichloroethylene; HRT-SE, Hit Reaction Time - Standard Error; PMA-R, Primary Mental Abilities – Reasoning.

^1^ Neuropsychological outcome scores were standardized as z-scores.

^2^ Data was calculated using a linear mixed model with cohort and individuals as random effects, visit as random slope, and adjusted for mother’s age at delivery, children’s sex and age on each visit, mother’s energy intake, maternal omega-3 supplementation during pregnancy, maternal IQ, rMed score (without fish), maternal education and maternal social class. The N of the sample was matched to the N of the sensitivity analysis model.

^3^ Fully adjusted model + maternal DDE serum levels at 12 weeks of pregnancy.

^4^ Lower scores indicate better performance.

^5^ Higher scores indicate better performance.

**Supplementary Table 12.** Association between large fatty fish consumption during pregnancy and offspring neuropsychological function up to 15 years of age, adjusted by maternal dichlorodiphenyltrichloroethane (DDT) serum levels at 12 weeks of pregnancy.

| **Neuropsychological outcome**^1^ |  | **Fully adjusted**^2^ | | | **Fully adjusted + DDT**^3^ | | |
| --- | --- | --- | --- | --- | --- | --- | --- |
|  |  | **β** | **95% CI** | ***p*-value** | **β** | **95% CI** | ***p*-value** |
|  | Maternal fish intake in the 1^st^ trimester |  |  |  |  |  |  |
| Attention^4^  ANT  (HRT-SE)  N = 1273 |  |  |  |  |  |  |  |
|  | Lowest tertile | Ref. | | | | | |
|  | Middle tertile | -0.13 | -0.24; -0.02 | 0.021 | -0.13 | -0.24; -0.02 | 0.019 |
|  | Higher tertile | -0.10 | -0.20; -0.01 | 0.030 | -0.11 | -0.20; -0.01 | 0.028 |
|  | Tertiles in continuous | -0.05 | -0.10; -0.01 | 0.023 | -0.06 | -0.10; -0.01 | 0.022 |
| (HRT mean) | Lowest tertile | Ref. | | | | | |
| N = 1273 | Middle tertile | -0.10 | -0.21; 0.01 | 0.062 | -0.10 | -0.21; 0.00 | 0.059 |
|  | Higher tertile | -0.07 | -0.17; 0.02 | 0.109 | -0.08 | -0.17; 0.02 | 0.102 |
|  | Tertiles in continuous | -0.04 | -0.08; 0.01 | 0.094 | -0.04 | -0.08; 0.01 | 0.088 |
| Working Memory^5^  N-back  (d2’)  N = 1179 |  |  |  |  |  |  |  |
|  | Lowest tertile | Ref. | | | | | |
|  | Middle tertile | 0.04 | -0.07; 0.15 | 0.480 | 0.04 | -0.07; 0.15 | 0.476 |
|  | Higher tertile | 0.04 | -0.06; 0.14 | 0.420 | 0.04 | -0.06; 0.14 | 0.420 |
|  | Tertiles in continuous | 0.02 | -0.03; 0.07 | 0.407 | 0.02 | -0.03; 0.07 | 0.407 |
| (d3’)  N = 1175 | Lowest tertile | Ref. | | | | | |
|  | Middle tertile | 0.12 | 0.01; 0.24 | 0.032 | 0.12 | 0.01; 0.24 | 0.032 |
|  | Higher tertile | 0.00 | -0.10; 0.10 | 0.928 | 0.00 | -0.10; 0.10 | 0.928 |
|  | Tertiles in continuous | 0.01 | -0.04; 0.06 | 0.820 | 0.01 | -0.04; 0.06 | 0.820 |
| Fluid Intelligence^5^  (PMA-R & Raven)  N = 995 | Lowest tertile | Ref. | | | | | |
|  | Middle tertile | 0.15 | 0.00; 0.29 | 0.047 | 0.15 | 0.00; 0.29 | 0.046 |
|  | Higher tertile | 0.13 | 0.00; 0.26 | 0.048 | 0.13 | 0.00; 0.26 | 0.046 |
|  | Tertiles in continuous | 0.07 | 0.00; 0.13 | 0.040 | 0.07 | 0.00; 0.13 | 0.039 |

ANT, Attention Network Test; CI, Confidence Interval; DDT, dichlorodiphenyltrichloroethane; HRT-SE, Hit Reaction Time - Standard Error; PMA-R, Primary Mental Abilities – Reasoning.

^1^ Neuropsychological outcome scores were standardized as z-scores.

^2^ Data was calculated using a linear mixed model with cohort and individuals as random effects, visit as random slope, and adjusted for mother’s age at delivery, children’s sex and age on each visit, mother’s energy intake, maternal omega-3 supplementation during pregnancy, maternal IQ, rMed score (without fish), maternal education and maternal social class. The N of the sample was matched to the N of the sensitivity analysis model.

^3^ Fully adjusted model + maternal DDT serum levels at 12 weeks of pregnancy.

^4^ Lower scores indicate better performance.

^5^ Higher scores indicate better performance.

**Supplementary Table 13.** Association between large fatty fish consumption during pregnancy and offspring neuropsychological function up to 15 years of age, adjusted by maternal polychlorinated biphenyls (PCBs) serum levels at 12 weeks of pregnancy.

| **Neuropsychological outcome**^1^ |  | **Fully adjusted**^2^ | | | **Fully adjusted + PCBs**^3^ | | |
| --- | --- | --- | --- | --- | --- | --- | --- |
|  |  | **β** | **95% CI** | ***p*-value** | **β** | **95% CI** | ***p*-value** |
|  | Maternal fish intake in the 1^st^ trimester |  |  |  |  |  |  |
| Attention^4^  ANT  (HRT-SE)  N = 1273 |  |  |  |  |  |  |  |
|  | Lowest tertile | Ref. | | | | | |
|  | Middle tertile | -0.13 | -0.24; -0.02 | 0.021 | -0.13 | -0.24; -0.02 | 0.020 |
|  | Higher tertile | -0.10 | -0.20; -0.01 | 0.030 | -0.11 | -0.20; -0.01 | 0.029 |
|  | Tertiles in continuous | -0.05 | -0.10; -0.01 | 0.023 | -0.06 | -0.10; -0.01 | 0.022 |
| (HRT mean) | Lowest tertile | Ref. | | | | | |
| N = 1273 | Middle tertile | -0.10 | -0.21; 0.01 | 0.062 | -0.10 | -0.21; 0.01 | 0.062 |
|  | Higher tertile | -0.07 | -0.17; 0.02 | 0.109 | -0.08 | -0.17; 0.02 | 0.108 |
|  | Tertiles in continuous | -0.04 | -0.08; 0.01 | 0.094 | -0.04 | -0.08; 0.01 | 0.094 |
| Working Memory^5^  N-back  (d2’)  N = 1179 |  |  |  |  |  |  |  |
|  | Lowest tertile | Ref. | | | | | |
|  | Middle tertile | 0.04 | -0.07; 0.15 | 0.480 | 0.04 | -0.07; 0.15 | 0.496 |
|  | Higher tertile | 0.04 | -0.06; 0.14 | 0.420 | 0.04 | -0.06; 0.14 | 0.440 |
|  | Tertiles in continuous | 0.02 | -0.03; 0.07 | 0.407 | 0.02 | -0.03; 0.07 | 0.428 |
| (d3’)  N = 1175 | Lowest tertile | Ref. | | | | | |
|  | Middle tertile | 0.12 | 0.01; 0.24 | 0.032 | 0.12 | 0.01; 0.24 | 0.037 |
|  | Higher tertile | 0.00 | -0.10; 0.10 | 0.928 | 0.00 | -0.10; 0.10 | 0.979 |
|  | Tertiles in continuous | 0.01 | -0.04; 0.06 | 0.820 | 0.00 | -0.05; 0.05 | 0.873 |
| Fluid Intelligence^5^  (PMA-R & Raven)  N = 995 | Lowest tertile | Ref. | | | | | |
|  | Middle tertile | 0.15 | 0.00; 0.29 | 0.047 | 0.15 | 0.00; 0.29 | 0.048 |
|  | Higher tertile | 0.13 | 0.00; 0.26 | 0.048 | 0.13 | 0.00; 0.26 | 0.049 |
|  | Tertiles in continuous | 0.07 | 0.00; 0.13 | 0.040 | 0.07 | 0.00; 0.13 | 0.041 |

ANT, Attention Network Test; CI, Confidence Interval; HRT-SE, Hit Reaction Time - Standard Error; PCBs, polychlorinated biphenyls; PMA-R, Primary Mental Abilities – Reasoning.

^1^ Neuropsychological outcome scores were standardized as z-scores.

^2^ Data was calculated using a linear mixed model with cohort and individuals as random effects, visit as random slope, and adjusted for mother’s age at delivery, children’s sex and age on each visit, mother’s energy intake, maternal omega-3 supplementation during pregnancy, maternal IQ, rMed score (without fish), maternal education and maternal social class. The N of the sample was matched to the N of the sensitivity analysis model.

^3^ Fully adjusted model + maternal PCBs (sum of 118, 153, 138, 180) serum levels at 12 weeks of pregnancy.

^4^ Lower scores indicate better performance.

^5^ Higher scores indicate better performance.

**Supplementary Table 14.** Corrected p-for-trends between prenatal nut, total seafood and large fatty fish consumption and offspring neuropsychological outcomes for multiple testing using the Benjamini-Hochberg false discovery rate.

| Neuropsychological outcome x  Exposure | Original *p*-value | Critical Value | Benjamini-Hochberg Adjusted *p*-value | Significance using an FDR of 0.05 |
| --- | --- | --- | --- | --- |
| HRT-SE x fatty fish | 0.004 | 0.003 | 0.035 | Yes |
| Fluid intelligence x fatty fish | 0.006 | 0.007 | 0.035 | Yes |
| d3’ x Nut | 0.007 | 0.010 | 0.035 | Yes |
| HRT x fatty fish | 0.032 | 0.013 | 0.107 | No |
| HRT-SE x Nut | 0.041 | 0.017 | 0.107 | No |
| d2’ x Nut | 0.043 | 0.020 | 0.107 | No |
| Fluid intelligence x Nut | 0.085 | 0.023 | 0.182 | No |
| d2’ x fatty fish | 0.222 | 0.027 | 0.377 | No |
| HRT x Nut | 0.226 | 0.030 | 0.377 | No |
| HRT-SE x seafood | 0.338 | 0.033 | 0.507 | No |
| d3’ x seafood | 0.492 | 0.037 | 0.626 | No |
| d3’ x fatty fish | 0.501 | 0.040 | 0.626 | No |
| HRT x seafood | 0.548 | 0.043 | 0.632 | No |
| Fluid intelligence x seafood | 0.632 | 0.047 | 0.677 | No |
| d2’ x seafood | 0.94 | 0.050 | 0.940 | No |

HRT-SE, Hit Reaction Time - Standard Error.
